# National and subnational burden and causes of anemia in China from 1990 to 2023: findings from the Global Burden of Disease Study 2023

**DOI:** 10.1186/s40779-025-00681-5

**Published:** 2025-12-15

**Authors:** Zheng Long, Ling-Ling Yu, Fan-Shu Yan, Pei-Pei Li, Li-Jun Wang, Mai-Geng Zhou, Bing-Xin Ji, Peng Yin

**Affiliations:** 1 Medical Affairs Office, Xuanwu Hospital, Capital Medical University, Beijing, 100053 China; 2 National Center for Chronic and Noncommunicable Disease Control and Prevention, Chinese Center for Disease Control and Prevention, Beijing, 100050 China; 3 Department of Hematology, Xuanwu Hospital, Capital Medical University, Beijing, 100053 China

**Keywords:** Anemia, Prevalence, Years lived with disability (YLD), Burden of disease, Trend

## Abstract

**Background:**

Anemia is a major global health problem. There were 89% of all anemia-related disabilities in developing countries. We aim to analyze the burden of anemia and its underlying causes in China from 1990 to 2023.

**Methods:**

Utilizing the data of the 2023 Global Burden of Disease (GBD 2023) study, this study analyzed the burden of anemia in China between 1990 and 2023. Then we analyzed the number and rate of anemia attributed to 16 underlying causes for all genders and ages. Drivers of change in prevalence and years lived with disability (YLD) numbers due to anemia were explored by decomposition analysis. And locally weighted regression was used to estimate the relationship between socio-demographic index (SDI) and age-standardized prevalence rate (ASPR) and age-standardized YLD rate due to anemia.

**Results:**

From 1990 to 2023, the ASPR and age-standardized YLD rate showed a downward trend among all anemia types (*P* < 0.05), and the ASPR and age-standardized YLD rate of anemia in females were higher than those in males. The highest number and rate of prevalence were found in mild anemia, and the highest number and rate of YLD were found in moderate anemia. As age increased, the prevalence and YLD rate of anemia increased, with a significant increase in females aged 20−54, in particular of moderate anemia. In 2023, the highest ASPR and age-standardized YLD rate among all anemia types were in the Northwestern regions. Compared to 1990, 31 provinces, Hong Kong, and Macao exhibited declines in both the ASPR and the age-standardized YLD rate for anemia. In China, most of the prevalent cases and YLD were attributable to dietary iron deficiency in 2023. The total prevalence of anemia decreased by 46.14% [95% uncertainty interval (UI) 27.54−61.02], of which age-specific rate, population growth, and population aging accounted for -77.32%, 21.33%, and 9.84%, respectively. A negative association between SDI and the ASPR and age-standardized YLD rate of anemia was shown in China.

**Conclusions:**

From 1990 to 2023, the burden of anemia in China has decreased but remained heavy among women of childbearing age, the elderly, and in the Northwestern region. Tailored prevention and control strategies should be strengthened to reduce the burden of anemia in high-risk areas.

**Supplementary Information:**

The online version contains supplementary material available at 10.1186/s40779-025-00681-5.

## Background

Anemia is a major global health problem. In 2021, the prevalence of anemia was 1.92 billion across the world, accounting for 5.7% of all years lived with disability (YLD) and was the third largest cause of disability globally [1]. Previous studies showed that the burden of anemia was higher in lower socio-economic developing regions [1, 2]. There were 89% of all anemia-related disability in developing countries [3]. Research on the burden of anemia in low- and middle-income countries is particularly important [4]. The number of anemia patients in China reached 136 million in 2021, accounting for almost 7.0% of all anemia cases globally [1], which indicates that the anemia burden in China cannot be ignored.

To reduce anemia burden, the World Health Organization (WHO) [5], the UN Sustainable Development Goals (SDGs) [6], and the Chinese National Nutrition Plan (2017−2030) [7] have set ambitious reduction targets. Global Nutrition Targets aim to cut anemia prevalence among reproductive-age women (15−49 years) by 50% by 2030 to meet SDGs 2 and 3 [8, 9], and Chinese National Nutrition Plan focuses on populations like children under 5, pregnant women, and older adults [7]. However, due to factors such as research design, disease definitions, population selection, risk factor distribution, geographical and cultural variations, epidemiological studies of anemia in China show significant heterogeneity. It is thus important to clarify recent changes in long-term trends and geographical distribution using comparable data.

Anemia has also been associated with multiple adverse outcomes, including poor birth outcomes [10], decreased work productivity [11], and impaired cognitive and behavioral development [12], especially in preschool children and reproductive-age women [4, 13, 14]. Furthermore, anemia can also be a risk factor for other diseases, such as chronic kidney disease (CKD) [15], tuberculosis [16], and type 2 diabetes [17]. It is important to understand the distribution of anemia-related diseases. In addition, previous studies have mostly focused on anemia in specific populations or regions, lacking a systematic and comprehensive analysis of the burden of anemia at the provincial level and its related causes across different age groups [18–20]. This study fills this gap by conducting an in-depth investigation into the burden of anemia at the national and subnational levels and its related causes across different age groups, providing a more valuable scientific basis for formulating targeted provincial anemia prevention and control strategies in China.

The Global Burden of Disease (GBD) study integrates multi-dimensional data through the system and adopts a standardized analysis framework, providing scientific and comprehensive methodological support for assessing the burden of anemia. The diagnosis of anemia is based on reduced hemoglobin concentration, adopting specific hemoglobin concentration thresholds established by the WHO, which vary by age, sex, and pregnancy status [1]. Anemia severity is classified into 3 grades: mild, moderate, and severe, each corresponding to distinct hemoglobin concentration ranges [1]. Therefore, to better understand the epidemiology of anemia in China and to provide more accurate scientific information for prevention and control, our study aimed to examine trends of prevalence and YLD due to anemia and its causes in China at both national and subnational levels from 1990 to 2023 using data from the GBD 2023.

## Methods

### Data sources

Data for this study were obtained from the GBD 2023, which provides comprehensive data on 375 diseases and injuries in 204 countries and territories from 1990 to 2023. Detailed methodologies were documented in previous publications [21–29]. The GBD 2023 data are publicly accessible via the Global Health Data Exchange (GHDx) websites (https://ghdx.healthdata.org and https://ghdx.healthdata.org/gbd-2023).

The estimation of the anemia burden in China for the GBD 2023 was informed by a comprehensive synthesis of data obtained through systematic reviews and collaborations. Following the standard GBD protocol for data inclusion, we identified relevant sources from the GHDx, scientific literature databases such as PubMed and Chinese academic journals (e.g., CNKI, Wanfang, et al.), and official national and provincial health statistical reports, including the China Health Statistical Yearbook [22, 23]. This process yielded a wide array of datapoints, encompassing population-based surveys with hemoglobin measurements, such as the China Health and Nutrition Survey and the China Chronic Disease and Risk Factor Surveillance, as well as data from hospital records and registries.

In line with the approach used for adjusting bias in administrative data and accounting for varying case definitions in other GBD components, we implemented a series of data processing steps [21, 22, 24]. Input data that reported on anemia prevalence and YLD using alternative hemoglobin thresholds or that was aggregated in broad age ranges were adjusted using the meta-regression Bayesian, regularised, trimmed tool to enhance comparability [21, 24]. Furthermore, data that were non-sex-specific or spanned wide age intervals were split into standard 5-year age and sex groups using the age-sex patterns derived from more detailed data sources, a process employed in the modelling of kidney failure and diabetes [23, 24].

The final dataset incorporated representative data at both the national and provincial levels. For locations or years with sparse data, the modelling framework leveraged information from data-rich areas and covariates, such as the Healthcare Access and Quality Index, to inform estimates, a strategy consistently applied across GBD 2023 studies to ensure robust and comparable results [21–29].

### Definitions

Anemia is defined by decreased blood concentration of hemoglobin [30, 31], including nutritional deficiencies, inflammation, inherited hemoglobin abnormalities, blood loss, and hemolysis [4, 32]. The GBD study provided estimates of unique, continuous distributions of hemoglobin concentrations (g/L) adjusted for elevation, with corresponding anemia prevalence and YLD across different severity levels. These estimates covered 204 countries and territories, 21 GBD regions, both sexes, and 25 age groups ranging from 0−6 d to ≥ 95 years [1]. Anemia severity (mild, moderate, and severe) was defined by specific hemoglobin concentration thresholds that varied by age, sex, and pregnancy status (Additional file 1: Table S1).

### Measurements

Prevalence and YLD were used to estimate the trends in the burden of anemia in China from 1990 to 2023. The burden of anemia was analysed across gender and various age groups, ranging from < 5 years to ≥ 95 years, including < 5 years, 5−9 years, 10−14 years, 15−19 years, 20−24 years, 25−29 years, 30−34 years, 35−39 years, 40−44 years, 45−49 years, 50−54 years, 55−59 years, 60−64 years, 65−69 years, 70−74 years, 75−79 years, 80−84 years, 85−89 years, 90−94 years, and ≥ 95 years.

### Statistical Analysis

The GBD 2023 methodology adopts DisMod-MR 2.1 to estimate the disease burden of non-fatal diseases [21–29]. The prevalence and YLD of anemia in 31 provinces, Hong Kong, and Macao in China during 1990−2023 were analyzed. To compare the different population structures during different periods and regions, we calculated the age-standardized rates (ASR) of anemia and their changes over 1990−2023, using the global age structures as the reference point [21–29]. The 95% uncertainty interval (UI) is a standard indicator used in GBD 2023, calculated by drawing 1000 times from the posterior distribution in the modeling process to address possible heterogeneity from sampling error and non-sampling variance [21–29]. All reported measures included point estimates and the corresponding 95% UI in this study. The percentage change in numbers and rates was calculated as the difference between the numbers and rates in 2023 and 1990, divided by the numbers and rates in 1990, representing the direction and magnitude of change over the past 34 years. Average annual percentage change (AAPC) was used to analyze the temporal trends in the anemia ASRs from 1990 to 2023.

GBD 2023 classifies diseases and injuries into 4 levels: Level 1 causes encompass 3 major categories, communicable diseases, maternal, neonatal diseases, and malnutrition, non-communicable diseases, and injuries; Level 2 causes have 22 diseases, classified based on epidemiological research needs or the main systems in which diseases occur; Level 3 causes have 176 diseases, divided according to the incidence frequency, severity of single diseases, impact on public health interventions, or commonalities of disease groups, serving as the core classification of the study; Level 4 causes include 174 diseases, being more detailed classifications. Then using data on the prevalence and YLD of the causes of anemia, we analyzed the number and rate of anemia attributed to 16 underlying causes (Level 3) for all genders and ages, including CKD, cirrhosis and other chronic liver diseases, dietary iron deficiency, endocrine, metabolic, blood, and immune disorders, gynecological diseases, hemoglobinopathies and hemolytic anemias, human immunodeficiency virus/acquired immunodeficiency syndrome (HIV/AIDS), inflammatory bowel disease, intestinal nematode infections, malaria, maternal disorders, other neglected tropical diseases, other unspecified infectious diseases, schistosomiasis, upper digestive system diseases, and vitamin A deficiency.

Then, using the method developed by Gupta [33], we decomposed variation in the numbers of prevalence and YLD of anemia from 1990 to 2023, using 3 explanatory components: change in the growth of the total population, shifts in the population structure by age, and changes in the anemia rates of prevalence and YLD. In brief, this method decomposes the changes in the number of anemia prevalence and YLD between 1990 and 2023 into 3 factors. First, the contribution of total population growth, defined as the impact of changes in total population size on anemia burden, under the assumption of unchanged age structure. Second, the contribution of shifts in population age structure refers to burden changes caused by alterations in age distribution while maintaining constant prevalence or YLD rates across age groups. Third, the contribution of changes in prevalence and YLD rates represents the effect of changes in disease prevalence levels after excluding demographic factors. By sequentially simulating scenarios where each factor acts independently, the method quantifies their relative contributions to total burden changes, providing evidence for identifying key driving factors and thereby facilitating the development of targeted intervention strategies.

Finally, we also estimated the relationship between socio-demographic index (SDI) and age-standardized prevalence rate (ASPR) and age-standardized YLD rate due to anemia through locally weighted regression and then compared the observed values with the expected values.

Analyses were conducted using R v4.0.4 (The R Foundation for Statistical Computing), SAS v9.4 (SAS Institute, Inc., Cary, NC), and the Joinpoint Regression Program v4.8.0 (1 April 2020).

## Results

### Overview

**Fig. 1:**
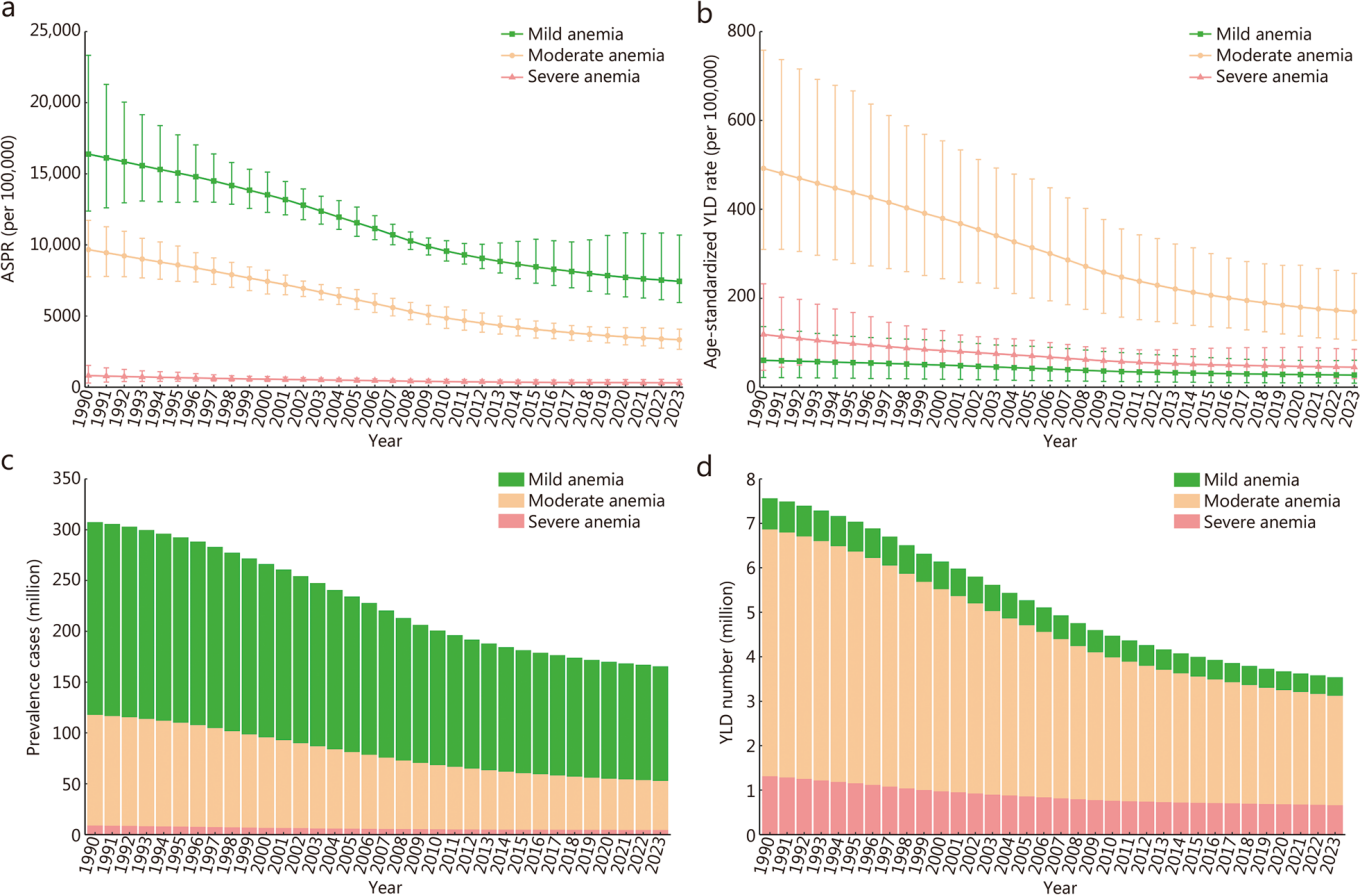
Annual trends in age-standardized prevalence rate (ASPR) (**a**), age-standardized YLD rate (**b**), all-age prevalence number (**c**), and all-age YLD number (**d**) for different types of anemia. YLD years lived with disability

In 2023, the number of prevalent cases of anemia in China was 165.60 million (95% UI 138.17−218.76), and the mild, moderate, and severe anemia cases accounted for 68.08%, 29.18%, and 2.74%, respectively. Anemia accounted for 3.54 million (95% UI 2.13−5.58) YLD in China, including 0.41 million (95% UI 0.14−0.92) from mild anemia, 2.46 million (95% UI 1.54−3.72) from moderate anemia, and 0.66 million (95% UI 0.27−1.28) from severe anemia (Table 1). Between 1990 and 2023, the ASPR and age-standardized YLD rate decreased for all types of anemia (AAPC < 0; Table 1 and Fig. 1). This burden was consistently higher among females compared to males. In 1990, the female ASPR was 28,763.20 per 100,000 (95% UI 23,315.68−36,542.19), notably higher than the male rate of 25,343.39 (95% UI 20,204.52−30,931.36). By 2023, while rates had declined in both groups, this disparity persisted: 12,759.80 (95% UI 10,093.46−17,752.83) in females vs. 9595.39 (95% UI 8072.91−11,832.77) in males (Additional file 1: Fig. S1). In 1990, the age-standardized YLD rate for females was 781.51 per 100,000 population (95% UI 505.08−1244.66), significantly higher than the male rate of 574.47 (95% UI 353.21−916.54). By 2023, rates had declined in both sexes, yet this gap persisted: 304.15 (95% UI 179.79−520.00) for females versus 185.66 (95% UI 109.33−313.39) for males (Additional file 1: Fig. S1). A consistent downward trend is evident in both the ASPR and age-standardized YLD rate for anemia across provinces and sexes since 1990. For instance, in Beijing, the ASPR decreased from 24,348.63 (95% UI 20,311.05−29,662.08) to 10,454.94 (95% UI 8012.68−16,142.50) per 100,000 population, while the age-standardized YLD rate fell from 592.87 (95% UI 389.10−893.85) to 224.14 (95% UI 119.50−412.87) per 100,000 between 1990 and 2023, as detailed in Additional file 1: Figs. S2-S10.

**Table 1:** Prevalence and YLD among all anemia types by gender in China between 1990 and 2023

Characteristics	Prevalence		YLD	
	Number (millions, 95% UI)	ASR (per 100,000, 95% UI)	Number (millions, 95% UI)	ASR (per 100,000, 95% UI)
Male				
Mild anemia				
1990	98.53 (75.47–131.33)	16,904.44 (12,984.71–22,823.98)	0.36 (0.13–0.79)	62.20 (22.41–135.92)
2023	54.54 (45.57–69.18)	6985.78 (5826.86–8901.59)	0.20 (0.07–0.44)	25.67 (8.52–56.06)
Change (%)	-44.64 (-61.25 to -24.71)	-58.67 (-71.95 to -42.35)	-44.84 (-61.40 to -24.80)	-58.73 (-71.99 to -42.31)
AAPC		-2.65 (-2.66 to -2.64)		-2.66 (-2.67 to -2.64)
Moderate anemia				
1990	41.74 (31.90–51.80)	7561.25 (5876.11–9224.18)	2.13 (1.32–3.32)	384.59 (236.59–593.51)
2023	16.14 (11.78–22.17)	2329.48 (1690.76–3227.00)	0.82 (0.50–1.35)	118.91 (69.30–187.59)
Change (%)	-61.33 (-73.37 to -34.37)	-69.19 (-78.47 to -50.07)	-61.43 (-73.62 to -34.43)	-69.08 (-78.42 to -50.00)
AAPC		-3.52 (-3.54 to -3.50)		-3.51 (-3.53 to -3.49)
Severe anemia				
1990	4.68 (1.94–8.36)	877.70 (343.39–1577.75)	0.68 (0.24–1.33)	127.67 (48.38–246.75)
2023	2.06 (0.92–3.83)	280.13 (132.59–535.01)	0.30 (0.13–0.59)	41.08 (18.23–82.06)
Change	-56.05 (-83.35 to 41.19)	-68.08 (-86.78 to 7.57)	-56.16 (-83.56 to 43.13)	-67.82 (-86.78 to 6.36)
AAPC		-3.41 (-3.43 to -3.39)		-3.39 (-3.41 to -3.36)
Female				
Mild anemia				
1990	91.11 (67.09–136.99)	16,021.98 (11,777.10–24,222.63)	0.34 (0.12–0.77)	59.03 (21.05–135.47)
2023	58.20 (42.05–96.14)	8019.48 (5921.06–13,034.76)	0.21 (0.07–0.46)	29.27 (9.31–63.10)
Change (%)	-36.12 (-61.20 to 1.32)	-49.95 (-69.74 to -20.78)	-36.85 (-61.26 to 1.13)	-50.42 (-69.70 to -20.74)
AAPC		-2.07 (-2.08 to -2.07)		-2.10 (-2.10 to -2.09)
Moderate anemia				
1990	67.15 (53.85–82.71)	11,969.59 (9637.11–14,626.33)	3.43 (2.18–5.32)	609.87 (389.72–944.67)
2023	32.18 (24.30–42.95)	4405.72 (3305.80–5989.65)	1.64 (0.99–2.63)	225.52 (135.99–368.08)
Change (%)	-52.07 (-67.14 to -31.00)	-63.19 (-74.38 to -48.70)	-52.09 (-67.09 to -31.13)	-63.02 (-74.30 to -48.59)
AAPC		-2.98 (-2.99 to -2.97)		-2.96 (-2.97 to -2.95)
Severe anemia				
1990	4.29 (1.31–8.55)	771.63 (236.01–1492.68)	0.63 (0.17–1.29)	112.60 (31.88–226.06)
2023	2.47 (0.82–5.06)	334.60 (124.73–711.91)	0.36 (0.11–0.78)	49.36 (17.48–105.46)
Change (%)	-42.30 (-83.46 to 123.66)	-56.64 (-86.25 to 50.20)	-42.11 (-83.49 to 122.71)	-56.17 (-86.22 to 50.54)
AAPC		-2.50 (-2.51 to -2.49)		-2.46 (-2.47 to -2.46)
Both				
Mild anemia				
1990	189.64 (143.45–268.14)	16,385.58 (12,375.56–23,326.66)	0.70 (0.25–1.57)	60.34 (21.57–136.18)
2023	112.74 (89.71–161.53)	7439.87 (5950.44–10,695.09)	0.41 (0.14–0.92)	27.24 (9.11–60.51)
Change (%)	-40.55 (-63.08 to -14.43)	-54.60 (-71.93 to -34.24)	-41.00 (-63.06 to -14.51)	-54.84 (-71.89 to -34.34)
AAPC		-2.37 (-2.38 to -2.36)		-2.38 (-2.39 to -2.37)
Moderate anemia				
1990	108.88 (86.42–133.64)	9669.21 (7763.43–11,721.46)	5.56 (3.48–8.59)	492.34 (309.74–757.73)
2023	48.33 (37.89–59.90)	3328.31 (2654.45–4070.85)	2.46 (1.54–3.72)	170.19 (105.50–255.96)
Change (%)	-55.62 (-69.03 to -36.88)	-65.58 (-74.93 to -52.85)	-55.67 (-69.06 to -37.02)	-65.43 (-74.94 to -52.72)
AAPC		-3.19 (-3.20 to -3.18)		-3.17 (-3.18 to -3.16)
Severe anemia				
1990	8.97 (3.33–17.16)	812.15 (289.52–1527.28)	1.31 (0.42–2.62)	118.41 (37.88–232.59)
2023	4.53 (1.80–8.27)	305.38 (136.35–547.24)	0.66 (0.27–1.28)	44.93 (19.27–85.03)
Change (%)	-49.48 (-83.24 to 58.04)	-62.40 (-85.99 to 14.40)	-49.45 (-83.32 to 60.34)	-62.06 (-85.94 to 15.88)
AAPC		-2.92 (-2.94 to -2.91)		-2.90 (-2.91 to -2.88)

*AAPC* average annual percent change, *ASR* age-standardized rate, *YLD* years lived with disability, *UI* uncertainty interval

**Fig. 2:**
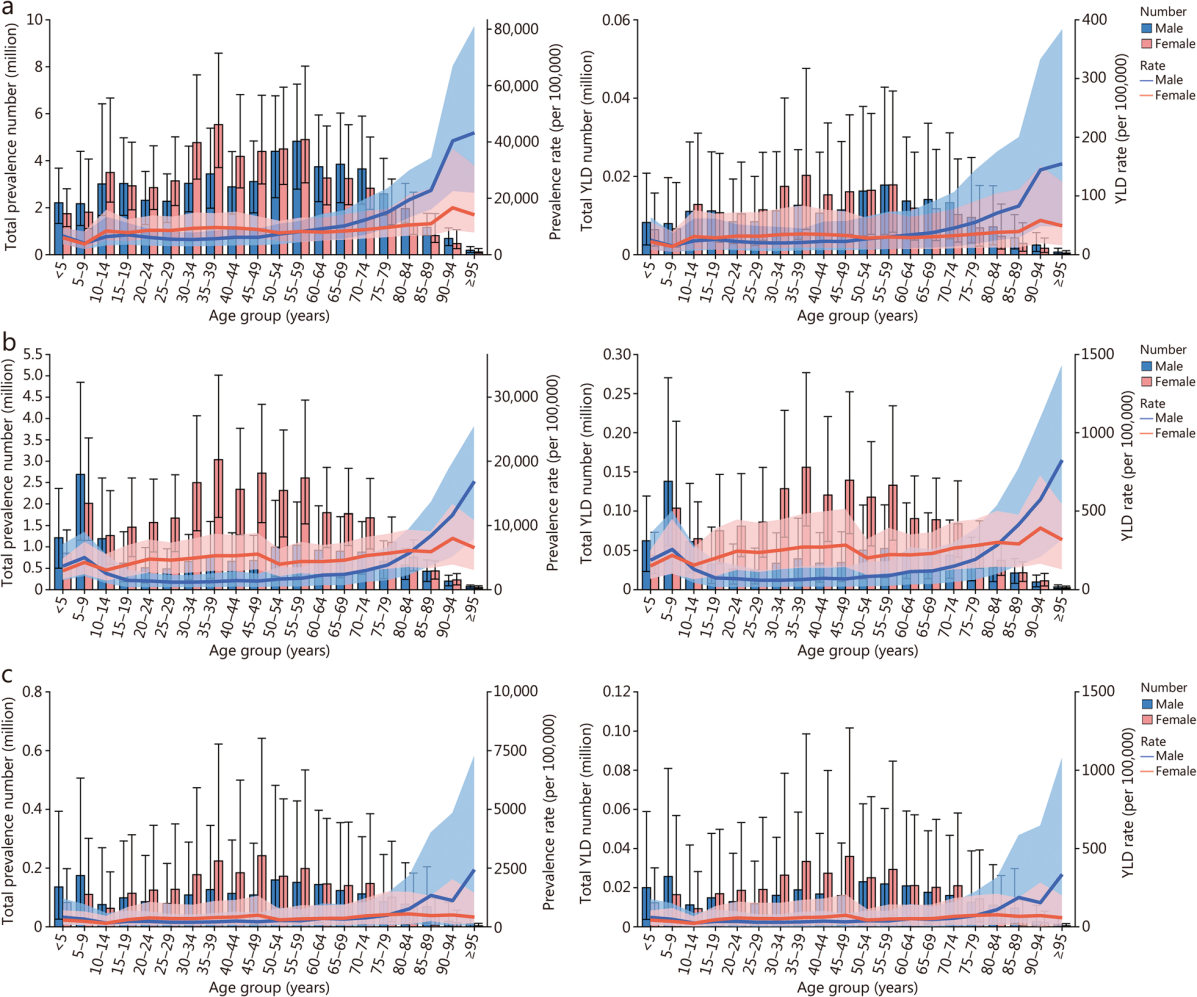
The numbers with prevalence rate and YLD rate for mild anemia (**a**), moderate anemia (**b**), and severe anemia (**c**) in China in 2023. YLD years lived with disability

The prevalence rate increased with age among all anemia types, with 2 marked inflection points at the age groups of 20−54 years [10,796.10 per 100,000 (95% UI 8,544.47−14,169.93)] and the ≥ 80 years [23,930.73 per 100,000 (95% UI 20,126.11−31,921.32)], especially in females in 2023. The trends of YLD in different age groups in 2023 were similar to prevalence (Fig. 2; Additional file 1: Fig. S11).

### Geographical Trends

In 2023, the highest ASPR and age-standardized YLD rates among all anemia types were in the Northwestern regions. For anemia, the highest ASPR and age-standardized YLD rate were observed in Ningxia [16,193.75 per 100,000 (95% UI 8256.34−49,926.34)] and Qinghai [365.83 per 100,000 (95% UI 132.74−956.42)], respectively. Compared to 1990, both the ASPR and the age-standardized YLD rate among all anemia types have decreased in 31 provinces, Hong Kong, and Macao of China in 2023 (Table 2; Additional file 1: Table S2).

**Table 2:** ASPR and age-standardized YLD rate with percentage changes of anemia for different types, in China, 1990−2023

Parameter	ASPR			Age-standardized YLD rate		
	1990 (per 100,000, 95% UI)	2023 (per 100,000, 95% UI)	Change (%, 95% UI)	1990 (per 100,000, 95% UI)	2023 (per 100,000, 95% UI)	Change (%, 95% UI)
Mild anemia						
**China**	16,385.58 (12,375.56–23,326.66)	7439.87 (5950.44–10,695.09)	-54.60 (-71.93 to -34.24)	60.34 (21.57–136.18)	27.24 (9.11–60.51)	-54.84 (-71.89 to -34.34)
Anhui	15,951.18 (11,923.34–22,155.62)	6578.57 (5432.30–8171.87)	-58.76 (-73.34 to -40.95)	58.76 (21.09–132.82)	24.10 (8.21–54.00)	-58.98 (-73.34 to -41.27)
Beijing	15,174.93 (11,826.50–20,606.05)	7178.38 (4610.71–12,725.38)	-52.70 (-69.85 to -24.74)	55.75 (20.06–120.01)	26.20 (8.26–68.79)	-53.01 (-69.77 to -24.95)
Chongqing	17,177.28 (12,797.42–24,519.11)	7712.45 (5527.94–14,272.87)	-55.10 (-72.91 to -26.07)	63.31 (22.63–142.29)	28.31 (8.97–69.56)	-55.29 (-72.62 to -26.06)
Fujian	15,723.27 (11,699.96–22,275.18)	6263.45 (4910.24–8334.63)	-60.16 (-74.71 to -41.96)	57.90 (20.83–128.75)	23.03 (7.66–52.30)	-60.22 (-74.79 to -42.26)
Gansu	18,305.66 (13,393.35–26,510.95)	9460.05 (6500.90–19,929.49)	-48.32 (-69.44 to 8.45)	67.44 (24.06–152.01)	34.60 (10.82–90.11)	-48.69 (-69.59 to 8.60)
Guangdong	15,494.15 (11,725.43–21,416.57)	6208.56 (5111.77–7528.74)	-59.93 (-74.84 to -42.32)	57.01 (20.46–129.03)	22.70 (7.71–50.06)	-60.19 (-74.81 to -42.41)
Guangxi	15,817.04 (11,793.11–22,393.93)	7051.26 (5599.30–9266.15)	-55.42 (-71.84 to -34.42)	58.25 (21.03–130.05)	25.89 (8.69–56.66)	-55.55 (-71.81 to -34.47)
Guizhou	18,698.34 (14,409.86–26,415.03)	8779.61 (6651.17–13,603.58)	-53.05 (-70.90 to -32.32)	68.81 (24.17–156.07)	32.29 (10.70–72.48)	-53.07 (-71.01 to -32.43)
Hainan	15,995.63 (10,808.89–25,106.63)	10,626.61 (4338.18–33,165.68)	-33.57 (-71.74 to 73.93)	58.97 (20.25–137.49)	38.41 (8.57–127.14)	-34.86 (-71.71 to 74.27)
Hebei	14,934.71 (11,230.31–20,712.62)	7719.36 (6403.68–9743.57)	-48.31 (-66.93 to -25.71)	54.98 (19.69–122.77)	28.24 (9.66–64.19)	-48.63 (-66.90 to -25.78)
Heilongjiang	15,961.90 (11,871.80–22,614.86)	7889.26 (6088.41–11,587.16)	-50.57 (-69.44 to -26.65)	58.77 (21.22–130.87)	29.03 (9.60–64.25)	-50.61 (-69.51 to -26.57)
Henan	15,723.53 (11,827.08–21,834.21)	7163.29 (5871.37–8754.23)	-54.44 (-72.22 to -33.70)	57.86 (20.79–130.54)	26.17 (9.10–58.24)	-54.78 (-72.19 to -33.37)
Hong Kong	13,178.59 (10,657.64–16,881.75)	8554.81 (6573.65–11,973.71)	-35.09 (-54.67 to -9.21)	48.45 (17.64–105.67)	31.39 (10.90–70.89)	-35.20 (-54.50 to -9.00)
Hubei	16,433.11 (12,225.62–23,317.22)	6684.33 (5523.07–8263.09)	-59.32 (-73.95 to -41.20)	60.52 (21.62–136.44)	24.49 (8.28–54.97)	-59.54 (-73.84 to -41.25)
Hunan	16,616.40 (12,368.13–23,330.37)	6880.42 (5759.21–8537.33)	-58.59 (-73.58 to -39.99)	61.19 (21.81–137.83)	25.21 (8.58–56.70)	-58.80 (-73.49 to -40.19)
Inner Mongolia	18,276.51 (13,456.41–26,220.17)	9627.73 (6512.77–19,789.65)	-47.32 (-68.93 to 8.23)	67.35 (24.12–149.73)	35.22 (11.02–95.41)	-47.72 (-68.59 to 8.13)
Jiangsu	15,374.93 (11,757.24–21,105.98)	6597.27 (5498.28–8032.16)	-57.09 (-72.32 to -39.40)	56.58 (20.19–126.94)	24.13 (8.20–53.78)	-57.36 (-72.39 to -39.28)
Jiangxi	16,324.85 (12,210.86–23,264.89)	6359.22 (5064.72–8196.51)	-61.05 (-75.22 to -43.00)	60.14 (21.62–135.63)	23.37 (7.87–52.05)	-61.15 (-75.25 to -43.18)
Jilin	16,976.85 (12,515.14–24,257.74)	8001.93 (5656.33–15,720.34)	-52.87 (-71.95 to -12.97)	62.56 (22.58–140.00)	29.37 (9.24–75.25)	-53.05 (-71.78 to -12.60)
Liaoning	14,969.19 (11,215.25–20,784.62)	7573.61 (5985.80–9963.14)	-49.41 (-67.83 to -25.75)	55.12 (19.97–122.37)	27.83 (9.41–61.48)	-49.50 (-67.76 to -25.44)
Macao	10,875.54 (8194.23–14,179.26)	5541.30 (4467.86–7081.62)	-49.05 (-65.53 to -28.02)	39.98 (14.81–86.34)	20.32 (6.71–44.58)	-49.17 (-65.57 to -27.53)
Ningxia	19,562.96 (13,524.44–31,532.45)	11,171.10 (4859.75–41,460.76)	-42.90 (-76.06 to 75.22)	72.18 (25.55–167.39)	40.06 (9.36–135.45)	-44.50 (-76.28 to 75.87)
Qinghai	14,574.10 (10,669.84–21,402.93)	9274.76 (4057.30–30,917.37)	-36.36 (-71.63 to 98.30)	53.61 (19.42–120.78)	33.47 (8.15–106.68)	-37.57 (-71.52 to 99.12)
Shaanxi	18,227.23 (14,132.50–25,654.22)	7886.55 (6080.13–11,751.92)	-56.73 (-72.70 to -38.78)	67.10 (23.67–151.80)	29.05 (9.62–65.35)	-56.70 (-72.67 to -38.94)
Shandong	15,536.46 (11,734.53–21,614.63)	7153.34 (5847.20–8706.67)	-53.96 (-71.87 to -32.75)	57.19 (20.50–129.10)	26.14 (9.00–57.74)	-54.30 (-71.69 to -32.57)
Shanghai	16,646.92 (13,806.27–21,152.31)	8589.76 (5832.01–17,396.88)	-48.40 (-67.79 to -0.62)	61.32 (21.64–131.48)	31.51 (9.63–79.05)	-48.62 (-67.82 to -0.78)
Shanxi	18,639.92 (14,447.24–25,771.87)	9535.41 (7257.76–14,727.44)	-48.84 (-67.45 to -27.29)	68.55 (24.27–152.15)	35.07 (11.64–78.59)	-48.84 (-67.39 to -27.26)
Sichuan	17,697.92 (12,932.21–25,236.34)	7870.81 (6489.81–9887.49)	-55.53 (-73.22 to -34.98)	65.19 (23.24–147.25)	28.76 (9.86–65.14)	-55.88 (-73.24 to -34.81)
Tianjin	14,959.42 (11,155.77–21,103.64)	8450.35 (4665.25–22,528.10)	-43.51 (-70.72 to 45.68)	55.07 (19.90–122.58)	30.75 (8.56–95.52)	-44.15 (-70.64 to 45.38)
Xizang	8918.62 (6584.56–12,473.50)	5339.49 (2916.34–9804.90)	-40.13 (-67.76 to 18.46)	32.88 (11.72–74.54)	19.66 (5.56–52.00)	-40.21 (-67.44 to 18.51)
Xinjiang	18,528.93 (13,489.71–26,729.00)	10,681.07 (6896.68–25,769.55)	-42.35 (-66.99 to 20.68)	68.31 (24.33–152.39)	38.81 (11.84–102.01)	-43.19 (-66.95 to 20.56)
Yunnan	18,302.01 (13,465.38–26,928.01)	8931.90 (6956.25–12,077.28)	-51.20 (-70.87 to -25.62)	67.42 (24.01–151.75)	32.82 (11.13–71.58)	-51.33 (-70.96 to -25.31)
Zhejiang	15,857.94 (12,156.55–21,869.06)	6603.19 (5340.14–8519.38)	-58.36 (-72.54 to -40.83)	58.40 (20.87–129.28)	24.24 (8.19–53.69)	− 58.49 (-72.40 to -41.17)
Moderate anemia						
**China**	9669.21 (7763.43–11,721.46)	3328.31 (2654.45–4070.85)	-65.58 (-74.93 to -52.85)	492.34 (309.74–757.73)	170.19 (105.50–255.96)	-65.43 (-74.94 to -52.72)
Anhui	9864.02 (7925.27–11,907.89)	2992.64 (2368.71–3689.33)	-69.66 (-78.21 to -58.14)	502.32 (316.13–767.41)	153.17 (94.89–227.51)	-69.51 (-78.32 to -58.00)
Beijing	8434.87 (7193.33–9648.68)	2961.07 (1887.23–4249.09)	-64.89 (-78.59 to -47.32)	429.24 (270.10–649.64)	151.49 (86.13–261.99)	-64.71 (-78.59 to -47.59)
Chongqing	10,830.90 (8674.89–13,013.25)	3344.07 (2584.55–4107.38)	-69.12 (-77.58 to -56.66)	551.41 (346.37–842.36)	170.99 (104.69–260.53)	-68.99 (-77.71 to -56.03)
Fujian	9082.22 (7213.73–11,012.57)	2741.38 (2130.87–3386.48)	-69.82 (-79.10 to -57.69)	462.30 (286.81–714.88)	140.21 (86.55–211.21)	-69.67 (-78.84 to -57.16)
Gansu	10,960.76 (8749.47–13,205.03)	4024.12 (3069.30–4925.14)	-63.29 (-73.93 to -47.64)	557.28 (353.96–848.20)	206.11 (122.83–330.63)	-63.02 (-74.01 to -48.00)
Guangdong	9354.88 (7502.29–11,372.48)	2766.98 (2109.57–3414.69)	-70.42 (-78.67 to -56.62)	476.48 (301.19–736.74)	141.43 (85.93–212.97)	-70.32 (-78.58 to -57.48)
Guangxi	8906.03 (7053.89–10,879.72)	3149.34 (2472.73–3891.30)	-64.64 (-75.33 to -50.78)	453.39 (282.33–702.34)	160.94 (99.55–240.80)	-64.50 (-75.12 to -50.29)
Guizhou	11,176.71 (8928.93–13,488.13)	3921.21 (3105.76–4807.99)	-64.92 (-74.81 to -51.41)	568.72 (359.96–886.75)	200.26 (124.01–299.70)	-64.79 (-74.50 to -50.98)
Hainan	8360.34 (6914.19–10,017.37)	3945.20 (1707.65–8928.06)	-52.81 (-78.94 to -3.48)	426.28 (266.16–659.84)	203.09 (83.40–474.03)	-52.36 (-78.78 to -3.52)
Hebei	8282.24 (6598.80–10,209.12)	3530.37 (2773.97–4344.48)	-57.37 (-69.77 to -40.27)	421.99 (264.74–658.35)	180.34 (110.98–269.40)	-57.26 (-69.63 to -39.56)
Heilongjiang	9010.04 (7126.49–11,007.74)	3578.45 (2828.89–4399.17)	-60.28 (-72.22 to -44.79)	458.69 (286.61–706.37)	182.82 (113.46–274.70)	-60.14 (-71.88 to -44.57)
Henan	9125.59 (7288.87–11,168.55)	3239.04 (2495.48–3991.08)	-64.51 (-74.48 to -48.96)	464.53 (294.47–716.91)	165.50 (100.89–246.05)	-64.37 (-74.09 to -48.76)
Hong Kong	7433.86 (5480.11–9583.13)	4740.82 (3148.28–7288.69)	-36.23 (-54.65 to -7.25)	378.59 (228.79–562.20)	241.53 (132.75–403.49)	-36.20 (-54.63 to -6.53)
Hubei	9800.78 (7849.11–11,894.79)	2963.58 (2354.09–3652.47)	-69.76 (-78.42 to -57.96)	499.24 (315.19–764.60)	151.65 (92.71–227.84)	-69.62 (-78.06 to -57.74)
Hunan	9664.74 (7717.30–11,762.58)	3027.83 (2369.63–3726.40)	-68.67 (-77.81 to -56.84)	491.99 (307.94–752.48)	154.76 (94.62–237.52)	-68.54 (-77.98 to -56.88)
Inner Mongolia	11,342.54 (9082.94–13,582.52)	4197.93 (3198.15–5133.84)	-62.99 (-73.38 to -47.89)	577.15 (367.56–878.01)	214.92 (127.29–341.11)	-62.76 (-73.39 to -47.77)
Jiangsu	9405.00 (7557.45–11,418.29)	3025.62 (2381.96–3713.98)	-67.83 (-76.75 to -54.77)	479.05 (303.91–738.77)	154.71 (95.23–231.06)	-67.70 (-76.37 to -54.72)
Jiangxi	9871.42 (7902.10–11,929.80)	2845.86 (2229.45–3511.21)	-71.17 (-79.58 to -60.48)	502.16 (315.38–769.56)	145.54 (91.01–217.77)	-71.02 (-79.43 to -60.55)
Jilin	9714.45 (7765.22–11,800.56)	3351.70 (2544.48–4128.27)	-65.50 (-75.67 to -50.98)	494.52 (311.97–761.54)	171.50 (103.32–273.65)	-65.32 (-75.37 to -50.64)
Liaoning	8471.35 (6709.59–10,403.65)	3527.43 (2792.16–4351.14)	-58.36 (-70.70 to -42.55)	431.47 (268.86–671.75)	180.29 (112.01–268.70)	-58.22 (-70.16 to -42.29)
Macao	5237.11 (4109.05–6688.53)	2495.47 (1928.67–3119.26)	-52.35 (-66.64 to -34.30)	266.88 (163.63–427.28)	127.58 (77.57–197.95)	-52.20 (-66.04 to -34.54)
Ningxia	11,554.93 (9681.17–13,668.35)	4488.83 (2415.04–9434.99)	-61.15 (-80.18 to -22.60)	587.70 (372.58–891.06)	230.74 (108.95–502.97)	-60.74 (-79.99 to -22.24)
Qinghai	9177.00 (7655.46–10,930.05)	4475.90 (1833.43–9466.18)	-51.23 (-80.90 to 0.11)	467.22 (290.14–718.73)	229.82 (85.22–552.47)	-50.81 (-81.01 to 0.22)
Shaanxi	10,634.88 (8482.39–12,858.52)	3410.91 (2649.12–4212.57)	-67.93 (-77.69 to -55.24)	541.60 (339.74–839.56)	174.40 (107.95–263.30)	-67.80 (-77.43 to -54.59)
Shandong	9244.98 (7382.59–11,304.61)	3296.54 (2519.12–4097.86)	-64.34 (-73.97 to -47.75)	471.04 (297.49–732.57)	168.71 (102.63–253.75)	-64.18 (-74.22 to -47.95)
Shanghai	10,220.04 (8658.95–11,723.34)	3910.81 (2810.41–5160.40)	-61.73 (-73.50 to -45.92)	520.49 (332.24–775.93)	200.42 (119.41–332.32)	-61.49 (-73.40 to -45.59)
Shanxi	11,795.45 (9477.65–14,142.42)	4482.94 (3565.03–5446.11)	-61.99 (-72.85 to -47.73)	601.41 (383.23–931.77)	229.27 (142.80–340.54)	-61.88 (-72.83 to -47.62)
Sichuan	10,169.76 (8119.68–12,362.33)	3427.72 (2618.38–4268.07)	-66.29 (-75.90 to -51.15)	517.57 (327.43–796.84)	175.12 (107.32–264.04)	-66.16 (-75.67 to -51.18)
Tianjin	8840.46 (7066.11–10,645.49)	3757.86 (2082.14–6077.88)	-57.49 (-77.08 to -30.83)	450.16 (286.12–699.19)	192.51 (103.66–383.08)	-57.24 (-76.82 to -31.46)
Xizang	4884.61 (3927.94–5953.43)	2942.41 (1289.80–6691.52)	-39.76 (-72.31 to 29.27)	248.43 (153.86–399.96)	150.75 (57.59–369.78)	-39.32 (-72.58 to 29.55)
Xinjiang	11,159.18 (8831.99–13,436.75)	4632.43 (3508.13–5829.73)	-58.49 (-70.42 to -39.77)	568.17 (357.76–867.80)	236.88 (141.03–377.31)	-58.31 (-70.33 to -39.72)
Yunnan	10,965.46 (8782.02–13,241.90)	4065.28 (3162.15–5011.56)	-62.93 (-74.14 to -48.43)	557.55 (351.46–844.79)	207.57 (127.56–307.75)	-62.77 (-73.98 to -47.93)
Zhejiang	9644.84 (7707.87–11,667.92)	2974.36 (2346.23–3664.51)	-69.16 (-77.98 to -58.30)	491.24 (309.50–757.65)	152.14 (94.66–227.61)	-69.03 (-78.18 to -58.26)
Severe anemia						
**China**	812.15 (289.52–1527.28)	305.38 (136.35–547.24)	-62.40 (-85.99 to 14.40)	118.41 (37.88–232.59)	44.93 (19.27–85.03)	-62.06 (-85.94 to 15.88)
Anhui	887.16 (323.92–1635.56)	279.41 (132.01–474.29)	-68.51 (-87.42 to 2.82)	129.25 (41.36–251.48)	41.18 (18.28–76.84)	-68.14 (-87.47 to 2.83)
Beijing	738.83 (345.01–1207.94)	315.48 (68.88–681.40)	-57.30 (-89.90 to 13.54)	107.88 (46.08–189.98)	46.45 (9.47–117.28)	-56.94 (-89.73 to 14.89)
Chongqing	973.54 (341.14–1864.65)	309.29 (109.85–611.15)	-68.23 (-91.12 to 6.67)	141.80 (45.29–280.60)	45.52 (13.78–97.61)	-67.90 (-90.93 to 7.41)
Fujian	748.49 (262.21–1421.13)	246.84 (101.75–453.87)	-67.02 (-89.25 to 12.74)	109.24 (33.92–211.37)	36.35 (12.92–70.72)	-66.73 (-89.29 to 16.16)
Gansu	893.27 (284.36–1755.62)	347.55 (110.13–716.47)	-61.09 (-90.27 to 43.20)	130.13 (37.67–257.18)	50.98 (14.67–117.71)	-60.82 (-90.38 to 48.76)
Guangdong	819.84 (293.32–1536.17)	264.91 (129.18–461.16)	-67.69 (-87.40 to 9.78)	119.39 (38.08–230.91)	39.04 (17.18–75.31)	-67.30 (-87.03 to 10.44)
Guangxi	719.39 (243.89–1375.29)	273.54 (108.87–501.73)	-61.98 (-87.46 to 26.84)	104.86 (34.22–205.70)	40.26 (14.99–76.65)	-61.60 (-87.60 to 30.27)
Guizhou	904.52 (293.69–1787.61)	325.57 (119.13–629.07)	-64.01 (-88.20 to 23.89)	131.76 (44.03–266.68)	47.82 (15.21–98.15)	-63.71 (-88.36 to 27.87)
Hainan	649.49 (220.54–1182.89)	471.25 (34.22–1931.51)	-27.44 (-93.36 to 179.79)	95.20 (29.75–192.93)	70.04 (4.59–307.80)	-26.44 (-93.51 to 182.18)
Hebei	670.53 (232.36–1249.60)	313.44 (144.63–538.82)	-53.26 (-82.78 to 56.85)	97.68 (31.05–189.98)	46.13 (19.27–85.43)	-52.78 (-82.50 to 57.92)
Heilongjiang	732.31 (245.64–1411.03)	308.70 (119.56–580.60)	-57.85 (-86.43 to 38.94)	106.72 (33.56–213.49)	45.28 (15.45–89.85)	-57.57 (-86.74 to 44.87)
Henan	756.44 (258.28–1434.02)	292.61 (147.91–489.21)	-61.32 (-85.10 to 37.20)	110.22 (33.29–214.32)	43.07 (19.46–81.44)	-60.93 (-85.52 to 36.51)
Hong Kong	689.74 (276.96–1282.11)	601.68 (195.13–1753.61)	-12.77 (-68.37 to 196.66)	100.65 (34.20–194.93)	87.84 (27.00–279.19)	-12.72 (-67.84 to 199.38)
Hubei	826.22 (286.62–1564.65)	264.15 (121.53–454.28)	-68.03 (-88.01 to 9.13)	120.49 (38.26–239.25)	38.87 (16.50–73.16)	-67.74 (-87.56 to 14.73)
Hunan	794.56 (278.56–1520.98)	264.39 (123.62–440.96)	-66.72 (-87.44 to 10.86)	115.73 (36.63–232.19)	38.92 (16.08–71.05)	-66.37 (-87.80 to 12.68)
Inner Mongolia	987.36 (322.59–1920.42)	376.35 (119.10–770.43)	-61.88 (-90.05 to 35.63)	143.80 (43.75–281.19)	55.22 (15.47–122.57)	-61.60 (-89.89 to 34.90)
Jiangsu	845.33 (319.35–1566.19)	287.63 (142.44–483.81)	-65.97 (-86.98 to 7.41)	123.26 (41.94–236.87)	42.36 (18.29–74.95)	-65.63 (-86.48 to 6.38)
Jiangxi	855.95 (298.95–1614.96)	257.05 (107.12–457.79)	-69.97 (-89.64 to 0.33)	124.95 (39.61–247.67)	37.71 (13.28–72.81)	-69.82 (-89.96 to 2.54)
Jilin	775.00 (254.68–1523.51)	291.16 (94.04–599.33)	-62.43 (-90.00 to 30.84)	112.94 (33.62–230.62)	42.72 (13.00–91.64)	-62.18 (-90.04 to 33.73)
Liaoning	714.09 (251.21–1358.42)	316.57 (129.47–569.87)	-55.67 (-85.05 to 47.38)	104.10 (33.59–204.54)	46.47 (16.28–88.59)	-55.36 (-85.02 to 55.01)
Macao	393.38 (149.43–718.23)	223.73 (97.04–373.27)	-43.13 (-78.59 to 73.05)	57.29 (19.83–112.74)	32.88 (12.48–59.01)	-42.61 (-78.75 to 79.14)
Ningxia	940.20 (287.86–1780.31)	533.81 (39.67–2114.08)	-43.22 (-95.16 to 89.63)	137.37 (40.35–275.66)	79.14 (6.14–347.15)	-42.39 (-95.10 to 90.01)
Qinghai	931.75 (352.40–1650.61)	691.42 (49.57–2954.17)	-25.79 (-93.57 to 186.71)	136.33 (47.15–265.82)	102.53 (6.62–427.74)	-24.79 (-93.49 to 190.92)
Shaanxi	847.99 (286.16–1676.26)	281.89 (105.73–539.57)	-66.76 (-89.92 to 13.35)	123.53 (41.69–250.72)	41.42 (13.56–84.39)	-66.47 (-89.91 to 18.60)
Shandong	796.83 (269.99–1522.91)	308.08 (151.42–527.47)	-61.34 (-84.95 to 35.57)	116.37 (36.21–229.14)	45.41 (20.69–85.39)	-60.98 (-85.06 to 37.84)
Shanghai	898.47 (395.05–1522.67)	422.40 (103.80–995.42)	-52.99 (-88.51 to 38.16)	131.32 (50.45–238.89)	62.33 (15.38–163.43)	-52.54 (-88.50 to 41.57)
Shanxi	1035.34 (510.63–1904.12)	386.33 (147.69–738.18)	-62.69 (-86.74 to -3.06)	151.04 (64.63–288.65)	56.83 (18.80–113.55)	-62.37 (-86.64 to -4.55)
Sichuan	790.14 (254.28–1546.72)	288.62 (124.65–528.57)	-63.47 (-87.41 to 38.63)	115.16 (33.62–233.39)	42.33 (16.99–84.31)	-63.24 (-86.98 to 42.76)
Tianjin	790.90 (311.03–1452.47)	464.22 (61.22–1518.05)	-41.31 (-91.68 to 78.10)	115.76 (41.03–227.68)	68.46 (9.37–242.85)	-40.86 (-91.64 to 80.92)
Xizang	541.11 (235.48–926.06)	514.19 (67.32–1811.80)	-4.97 (-85.50 to 208.29)	78.76 (31.81–148.70)	76.48 (9.38–263.22)	-2.89 (-85.66 to 218.82)
Xinjiang	938.40 (299.91–1812.41)	409.06 (119.18–851.84)	-56.41 (-88.88 to 51.25)	136.75 (39.97–278.63)	60.02 (15.96–138.22)	-56.11 (-88.55 to 50.65)
Yunnan	878.65 (273.60–1702.91)	337.89 (124.84–632.38)	-61.54 (-87.73 to 41.24)	127.82 (37.29–260.13)	49.47 (16.53–105.91)	-61.29 (-87.55 to 49.96)
Zhejiang	858.55 (321.73–1587.69)	275.78 (121.28–481.36)	-67.88 (-87.72 to -3.49)	125.26 (41.15–238.92)	40.54 (15.47–75.57)	-67.63 (-87.72 to 0.67)

*ASPR* age-standardized prevalence rate, *YLD* years lived with disability, *UI* uncertainty interval

### Underlying Causes

**Fig. 3:**
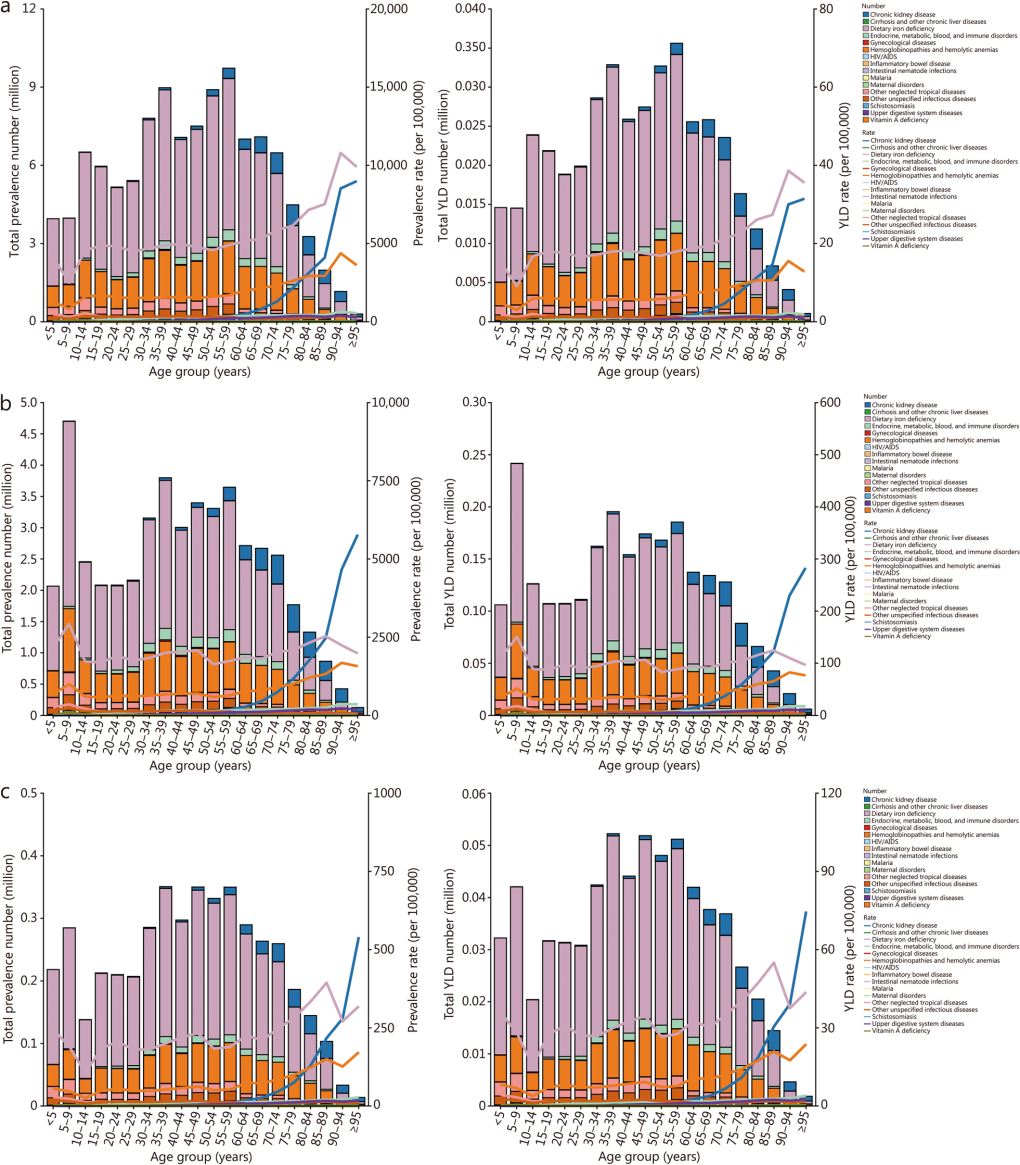
Number and rate for mild anemia (**a**), moderate anemia (**b**), and severe anemia (**c**) per 100,000 population attributable to each underlying cause by age in China in 2023. YLD years lived with disability, HIV/AIDS human immunodeficiency virus/acquired immunodeficiency syndrome

In 2023, the prevalence rate [6954.11 per 100,000 (95% UI 5742.06−8774.55)] and the YLD rate [147.58 per 100,000 (95% UI 84.03−245.51)] of anemia that was attributable to dietary iron deficiency were higher than all other causes, which were also found in most age groups, among all anemia types. The prevalence rate attributable to CKD started to increase in the 50−54 years and reached its peak in the ≥ 95 years. The rates in these groups were 315.27 per 100,000 (95% UI 204.62−541.84) and 15,304.65 per 100,000 (95% UI 9772.86−24,408.58) (Fig. 3; Additional file 1: Fig. S12).

### Decomposition Analysis

**Fig. 4:**
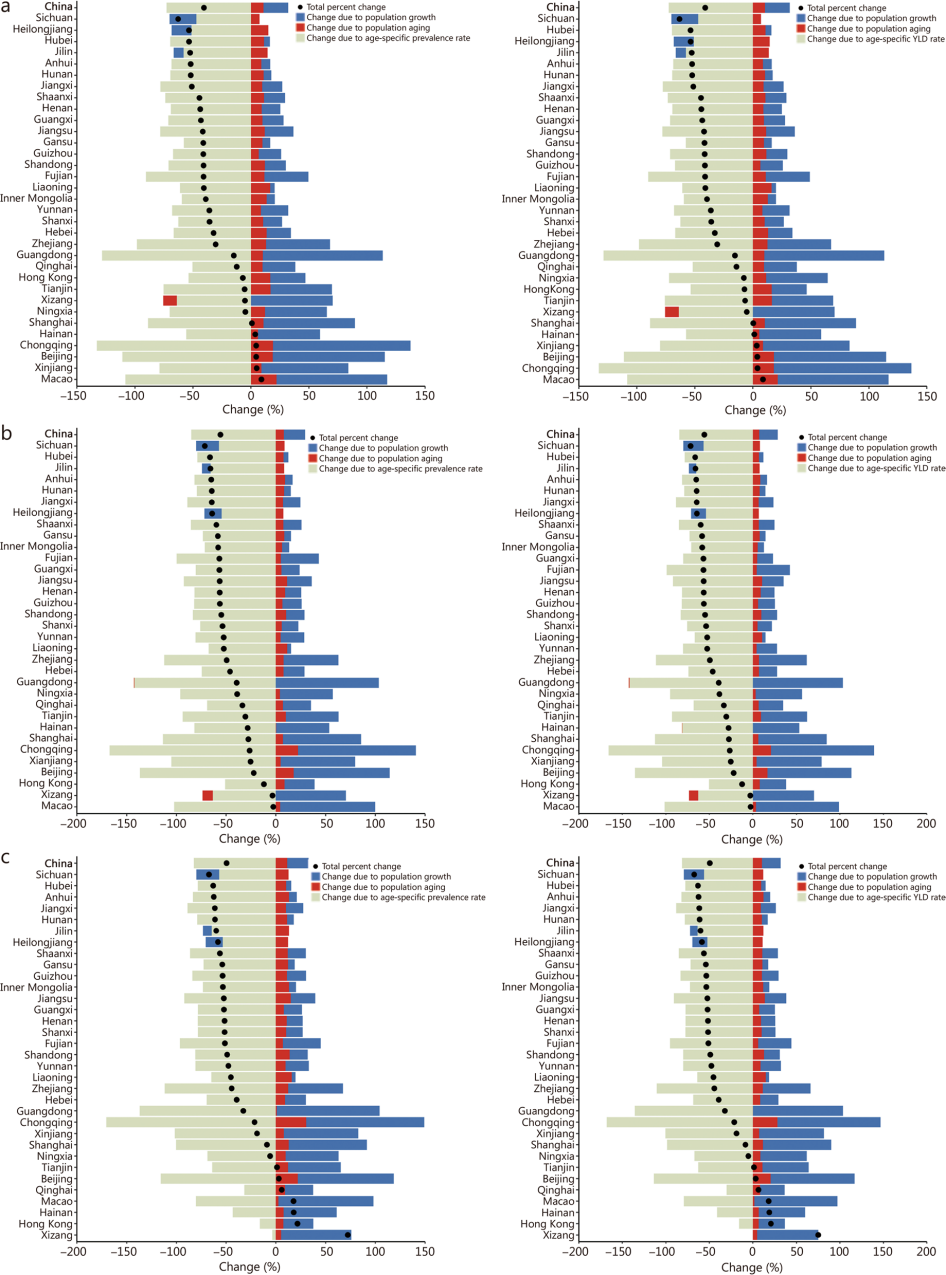
Increment for mild anemia (**a**), moderate anemia (**b**), and severe anemia (**c**) prevalence and YLD due to the changes in population growth, population aging, and age-specific prevalence rate in China from 1990 to 2023. YLD years lived with disability

The decomposition analysis of increment of numbers of prevalence and YLD among all anemia types between 1990 and 2023 are showed in Fig. 4. Compared with 1990, the total number of prevalence due to anemia in China in 2023 decreased by 46.14% (95% UI 27.54−61.02), of which the change in prevalence rate accounted for -77.32%, population growth accounted for 21.33%, and population aging accounted for 9.84% (Additional file 1: Fig. S13). Both the prevalence and YLD due to anemia declined across most provinces, but there were still differences. The decomposition analysis of YLD was similar to prevalence, population growth, and population aging were key drivers of the increase in YLD (Fig. 4).

### Association with the SDI

**Fig. 5:**
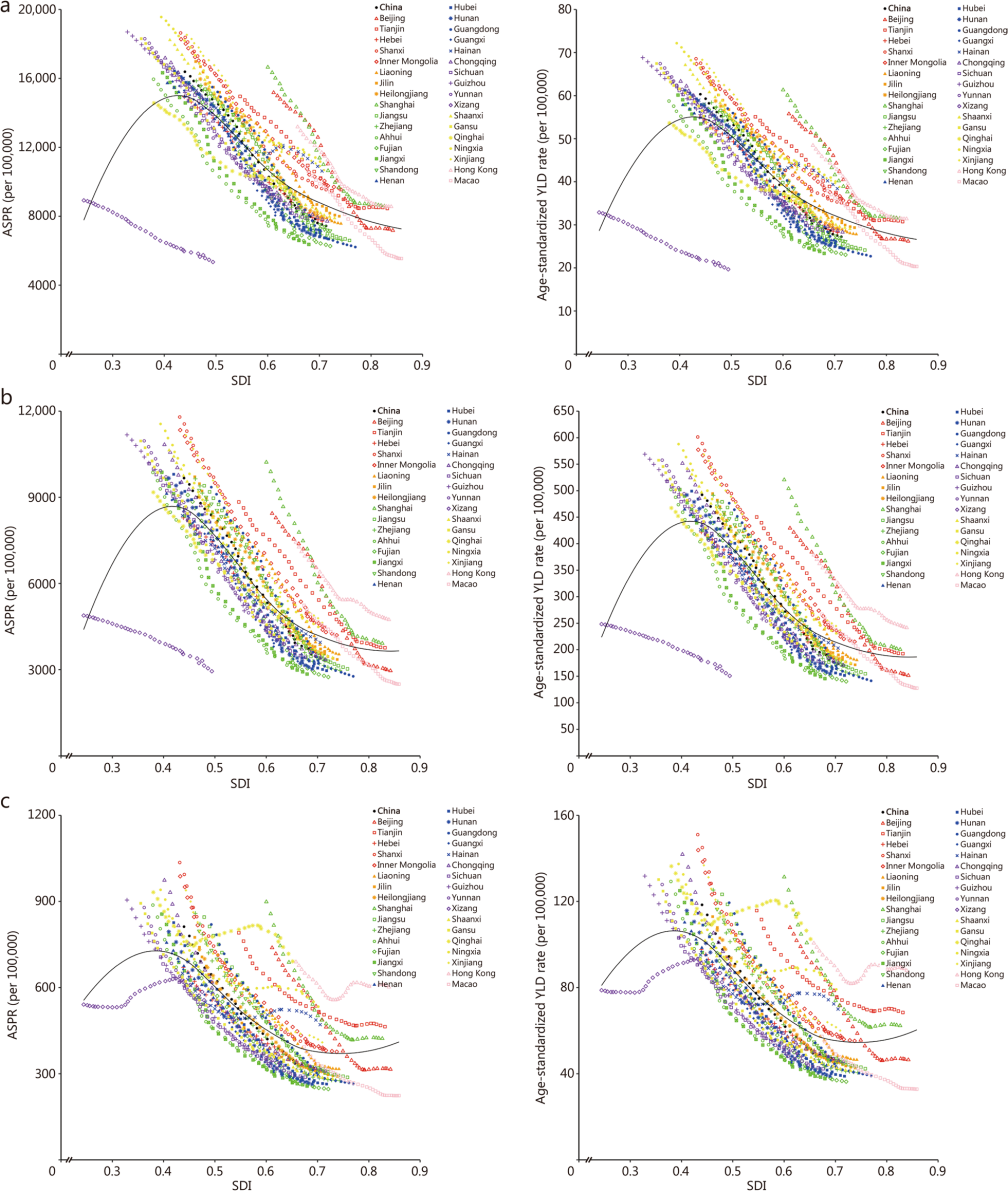
The association between SDI and the age-standardized prevalence rate (ASPR) and age-standardized YLD rate for mild anemia (**a**), moderate anemia (**b**), and severe anemia (**c**) in China. Black line locally weighted regression curve. YLD years lived with disability, SDI socio-demographic index

Overall, there was a negative association between SDI and the ASPR and age-standardized YLD rate of anemia among all anemia types in China, suggesting that the burden of anemia was higher in provinces with lower SDI. However, Xizang might experience underreporting, misclassification, or sampling bias of anemia cases due to its geographical environment (with high altitude being an important component), scattered population distribution, and challenges in grassroots health data collection. Its anomalously low prevalence and YLD rate due to anemia might reduce the overall trend fit for adjacent SDI categories (Fig. 5; Additional file 1: Fig. S14).

## Discussion

The prevalence of anemia and YLD rate decreased remarkably in China from 1990 to 2023. The prevalence and YLD rate of anemia increased with age and were higher in females than in males, with a significant elevation in the female population aged 20 to 54 years. The highest number and rate of prevalence were found in mild anemia, and the highest number and rate of YLD were found in moderate anemia. The 31 provinces, Hong Kong, and Macao had a reduction in ASPR and age-standardized YLD rate. However, the prevalence and YLD rate of anemia in Northwestern provinces were higher than in other regions during the study period. Most of the prevalent cases and YLD were attributable to dietary iron deficiency. There was a negative association between SDI and the ASPR, and the age-standardized YLD rate of anemia in China.

In 2023, the ASPR of anemia in China was 11,073.56 per 100,000, lower than the global and the East Asian. However, it remained higher than that of developed countries such as the United States of America and the United Kingdom, suggesting that there is still considerable room for improvement in anemia prevention and control in China [34]. Meanwhile, compared to other populous countries, the ASPR due to anemia in China was substantially lower than that of India, Indonesia, and Pakistan [1, 34]. China’s SDI level rose, reflecting nearly 3 decades of progress in education, income, and birth control, which has directly reduced the risks of malnutrition and maternal anemia [20, 35]. Additionally, China has successfully controlled tropical and parasitic diseases like malaria, hookworm disease, and schistosomiasis, which are major causes of global anemia [1]. Sub-Saharan Africa and South Asia have seen high rates of anemia due to the prevalence of these diseases [1], while China has significantly reduced infection-related chronic blood loss and inflammatory anemia through large-scale deworming programs and malaria elimination initiatives [36, 37]. In addition, China has implemented numerous policies to improve national nutrition, such as the National Nutrition Plan (2017−2030) [7] and Yingyangbao [38], which focuses on impoverished areas. However, China’s SDI has not yet reached the level of developed countries, indicating that there are still some rural and remote areas with unbalanced nutrition and insufficient medical resources [39, 40]. China’s aging population is becoming increasingly severe [41], and the high incidence and prevalence of chronic diseases such as CKD and diabetes [42, 43] might have contributed to the difference between China’s anemia burden and that of developed countries. The transnational nature of contemporary health issues necessitates strengthened international cooperation in the health sector. Therefore, fostering such collaboration is vital to build more resilient global health systems and facilitate the advancement of our common well-being goals.

The prevalence and YLD rate of anemia decreased gradually between 1990 and 2023, which might be related to nutrition improvement, better access to healthcare services, and improvements in the prevention and treatment of anemia. A previous study showed that the low-weight malnutrition rate among elderly people aged 60 and above in China dropped from 6.1% in 2012 to 3.8% in 2018 [44]. Nonetheless, the aging population, population growth, and enhanced survival rates of patients with chronic conditions like CKD [15] and hemoglobinopathies [45] indicate that anemia continues to pose a significant health burden in China.

The highest number and rate of prevalence were found in mild anemia, and the highest number and rate of YLD were found in moderate anemia in this study. This finding can be attributed to the severity-based anemia disability weights. Thus, perhaps more YLD could be avoided if moderate/severe anemia prevention is prioritized, such as iron supplements [46]. Mild anemia is a prevalent condition often overlooked, with many individuals unaware of its severity. The diagnostic criterion for mild anemia is defined as hemoglobin levels below the normal range but above 110 g/L (pregnant women and children aged 1 month to 4 years: above 100 g/L) in this study (Additional file 1: Table S1), encompassing a vast number of patients within this category. Moderate anemia might lead to more pronounced health issues in daily life for patients, such as fatigue, palpitations, and others, thereby contributing to higher YLD [4]. This impact is particularly evident in specific populations, including the elderly and those with chronic illnesses [1, 2, 47]. Therefore, there is a need for enhanced interventions targeting mild anemia in the future to prevent its progression to moderate or severe anemia.

Consistent with previous studies [1, 2, 10], the anemia prevalence and YLD rate in females were higher than those in males. It might be explained by the special physiological structure of females, including menstrual bleeding and pregnancy-related complications during the reproductive years, which can lead to depletion of iron stores, therefore necessitating higher iron intake [40]. This was also confirmed by this study, where the prevalence and YLD rate of anemia were significantly higher in females aged 20 to 54 years. In addition, the difference in dietary structure between males and females might also lead to the difference in anemia burden. In 2016−2017, the average daily intake of livestock meat for males was higher than that for females in all age groups [48]. Finally, influenced by the trend of “thin is beautiful”, females might pay more attention to their body shape, so they might diet to lose weight and leading to anemia.

Anemia among children < 5 years is a major public health problem; 46−66% of children < 5 years were affected by anemia in developing countries [3, 49]. Asian and African populations were the major contributors to a high burden of anemia [49]. Factors such as low birth weight, malnutrition, socioeconomic disadvantages, household food insecurity, breastfeeding duration, inadequate dietary iron intake, limited maternal education, diarrhea, fever, poverty, inadequate sanitation and hygiene, a monotonous diet, parents’ educational levels, and maternal anemia are frequently implicated in anemia among children under 5 years old [50–52]. Interestingly, we found a higher prevalence and YLD in boys aged < 5 years than in girls. Male infants and young children might have lower iron stores, which may put them at higher risk of iron deficiency anemia [53]. Therefore, the future of anemia publicity and education should also pay attention to male children.

Our study also found that the prevalence and YLD rate increase with increasing age. The elderly often suffer from a variety of chronic diseases [54], and the disease may cause damage to the hematopoietic system, leading to the occurrence of anemia. In addition, the long-term use of various drugs to treat chronic diseases, such as aspirin, diuretics, and other long-term medications, may inhibit the bone marrow hematopoietic function [55]. However, the prevalence (> 70 years) and YLD (> 80 years) rates were higher in older males than in females, which was consistent with previous research [56]. CKD was found to be one of the leading causes of anemia in the elderly, and males had a greater decrease in hemoglobin concentration than females at reduced levels of renal function [47].

The ASPR and age-standardized YLD rate of anemia in China declined between 1990 and 2023. However, provinces in the Northwestern regions, such as Qinghai, Ningxia, and Xinjiang, exhibit significantly higher rates than economically developed provinces in the East, such as Jiangxi, Fujian, and Guangdong. This disparity may be attributed to variations in economic and cultural conditions, dietary habits, natural geographical environments, and unequal distribution of medical resources across regions [48, 57–60]. Firstly, differences in economic development levels may influence the burden of anemia [57]. Our study also found that there was a negative association between SDI and the burden of anemia. Under the impact of the global economic crisis, anemia prevalence has increased in rural and impoverished areas [58]. Secondly, variations in dietary structures and habits can directly impact iron absorption, thereby contributing to the differences in anemia burden. Dietary habits vary significantly across different regions of China, which might contribute to differences in the burden of anemia. The Eastern and Southern regions have higher seafood consumption, while the Southwestern region has a higher intake of red meat and lower fruit consumption, and in the Northern region, although dairy consumption is higher, vegetable intake is lower [59]. In addition, vitamin C is an effective enhancer of iron absorption. In 2015, adults aged 18−59 in Eastern China consumed 275.9 g/person every day of fresh vegetables and 41.5 g/person every day of fruits, higher than those in Eastern (266.1 g/person every day and 32.6 g/person every day) and Western regions (220.1 g/person every day and 29.0 g/person every day) [48]. Thirdly, unequal distribution of medical resources leads to disparities in anemia diagnosis and treatment levels across different regions. Remote and impoverished areas in the West may lack specialized medical services and diagnostic tools. The Western regions of our country (such as Qinghai, Ningxia, and Xinjiang) face prominent contradictions in the distribution of medical resources due to harsh geographical conditions (the Tibetan Plateau, widespread deserts), sparse population, and lagging economy [60]. For example: 1) the number of hospitals (especially secondary and tertiary hospitals) and bed capacity is far lower than in the Eastern regions, concentrated mainly in a few core cities, with weak primary healthcare services at the county and rural levels; 2) the sparse road network results in longer travel times for residents seeking medical care, often exceeding 60 min in some remote areas; and 3) the “hospital capacity-to-population ratio” in the Western regions is significantly low, leading to an imbalance between supply and demand, and a large disparity in medical equity. In the future, these areas need to become key regions for optimizing resource allocation. Fourthly, varying population age structures and gender ratios play a role, as young women and children are high-risk groups for anemia [1, 2, 4]. Therefore, the prevention and control of anemia should pay more attention to the poor areas in western China.

It’s worth noting that the prevalence and impact of anemia might vary across different regions and populations. This study found that dietary iron deficiency was the prominent cause of anemia in China, mirroring similar patterns observed worldwide [1]. Given the significance of these findings, it is indicated that the pivotal role of iron supplementation in addressing anemia among specific populations, such as surgical patients, pregnant women, and children [61–63]. Furthermore, our study also found that the burden of anemia resulting from CKD progressively increases with advancing age in China. Anemia is a prevalent complication of CKD, significantly impacting quality of life, work efficiency, and treatment outcomes [63]. A previous study found that CKD patients with anemia are associated with higher mortality rates, hospitalizations, and progression of CKD globally, and these risks escalate with the severity of anemia [64]. As CKD progresses tend to intensify with age, further elevating the probability of anemia [65]. Notably, China faces unique challenges in addressing these issues. The rapid aging of the Chinese population might exacerbate the anemia burden. Therefore, future efforts in China should prioritize addressing anemia, particularly in elderly CKD patients, not only to mitigate anemia burden but also to align with global best practices and improve overall health outcomes.

Our study found that the contribution of the 3 types of causes to the change in anemia prevalence and YLD is quite different. Due to the development of urbanization and migration, the contribution of population growth to the increase in prevalence and YLD number is relatively higher in the developed areas, such as Chongqing, Guangdong, Beijing, Macao, and Shanghai. However, the contribution of population growth in Jilin, Heilongjiang, and Sichuan had a positive effect on the decrease of anemia prevalence and YLD number. We found that age-specific rates had a positive effect on the decrease of anemia prevalence and YLD number, and population aging had a positive effect on the increase of anemia prevalence and YLD number.

Based on this study, we propose the following recommendations to reduce the burden of anemia in China. First, develop differentiated intervention measures for key populations, such as women aged 20−54 and the elderly. Second, pay attention to regional disparities in anemia burden, particularly in Northwestern provinces, and allocate prevention and control resources evenly. Balance general prevention for mild anemia with targeted treatment for moderate anemia. Third, address dietary iron deficiency, the primary cause of anemia, by promoting iron-rich diets, food fortification, and public nutrition education. Fourth, given the negative correlation between SDI and anemia-related indicators, continuously promote social development in economically underdeveloped areas, improve education levels, and enhance access to health services. Focus on key populations, address regional differences, strengthen nutritional interventions, and implement comprehensive policies in conjunction with socioeconomic development.

Our study also has some limitations. First, the data sources for estimating nonfatal outcomes related to anemia are sparse and heterogeneous and rely primarily on small-scale population-based surveys. Furthermore, it is noteworthy that the longitudinal estimates of anemia prevalence and YLD in GBD 2023 do not stem directly from anemia incidence itself, but rather are derived from models that incorporate multiple covariates. Some of these covariates may directly impact hemoglobin concentration (such as malaria and hemoglobin variants), others may indirectly influence anemia (like contraceptives), and still others reflect upstream health conditions. In addition, there might be problems such as under-reporting in Western regions (e.g., Xizang), resulting in low prevalence and YLD rate of anemia. Addressing anemia is a health priority, and although the strength of the GBD study lies in its use of Bayesian meta-regression modeling to generate optimal estimates in situations with limited data [21–29], our present results should be treated with caution. Second, our study did not investigate anemia-related risk factors owing to the lack of attributable risk factors for anemia in the GBD 2023 study. Third, our study was unable to further analyze the differences in the burden of anemia between urban and rural areas in China. Given the significant impact of socio-economic disparities on disease burden, we further explored the relationship between SDI and ASPR and age-standardized YLD rate due to anemia, and found that SDI was significantly negatively correlated with anemia burden, which indicates that the burden of anemia is heavier in areas with weak economic development. Last, our study underscores the need for greater attention to the burden of anemia across diverse populations, particularly in Western provinces, children below the age of 5 years, pregnant women, and older adults. Conducting more real-world studies is essential to validating our findings.

## Conclusions

From 1990 to 2023, the burden of anemia in China has decreased but remained heavy among women of childbearing age, the elderly, and in Northwestern provinces. More efforts should be made to develop tailored prevention and control strategies aiming to reduce the prevalence and YLD from anemia in high-risk populations, especially in children below the age of 5 years, pregnant women, older adults, and western regions.

## Supplementary Information

**Additional file 1**. **Table S1** Hemoglobin concentration thresholds (g/L) for classification of anemia severity by stratification variables: sex, age, and pregnancy status. **Table S2** ASPR and age-standardized YLD rate with percentage changes of anemia in China, 1990−2023. **Fig. S1** Annual trends in ASPR and age-standardized YLD rate for anemia. **Fig. S2** Annual trends in ASPR and age-standardized YLD rate for mild anemia (a), moderate anemia (b), and severe anemia (c). **Fig. S3** Annual trends in age-standardized prevalence rate (ASPR) for anemia in China. **Fig. S4** Annual trends in age-standardized YLD rate for anemia in China. **Fig. S5** Annual trends in age-standardized prevalence rate (ASPR) for mild anemia in China. **Fig. S6** Annual trends in age-standardized YLD rate for mild anemia in China. **Fig. S7** Annual trends in age-standardized prevalence rate (ASPR) for moderate anemia in China. **Fig. S8** Annual trends in age-standardized YLD rate for moderate anemia in China. **Fig. S9** Annual trends in age-standardized prevalence rate (ASPR) for severe anemia in China. **Fig. S10** Annual trends in age-standardized YLD rate for severe anemia in China. **Fig. S11** The numbers with prevalence rates and YLD rates of anemia in China in 2023. **Fig. S12** Number and rate due to anemia per 100,000 population attributable to each underlying cause by age in China in 2023. **Fig. S13** Increment in anemia prevalence and YLD due to the changes in population growth, population aging, and age-specific prevalence rate in China from 1990 to 2023. **Fig. S14** The association between SDI and the age-standardized prevalence rate (ASPR) and age-standardized YLD rate of anemia in China.

## Data Availability

Research materials will be made available to other researchers at the reasonable request of the corresponding author.

## Abbreviations

AAPC: Average annual percentage change
AIDS: Acquired immunodeficiency syndrome
ASPR: Age-standardized prevalence rate
ASR: Age-standardized rate
CKD: Chronic kidney disease
GBD: Global burden of diseases
GHDx: Global Health Data Exchange
HIV: Human immunodeficiency virus
SDGs: Sustainable Development Goals
SDI: Socio-demographic index
YLD: Years lived with disability
