# Supplementary material for: National and subnational burden and causes of anemia in China from 1990 to 2023: findings from the Global Burden of Disease Study 2023
Source: Mil Med Res. 2025 Dec 15;12:91. doi: 10.1186/s40779-025-00681-5 (PMC12703925; doi:10.1186/s40779-025-00681-5)
Supplement: Supplementary file 1 — Additional file 1. Table S1 Hemoglobin concentration thresholds (g/L) for classification of anemia severity by stratification variables: sex, age, and pregnancy status. Table S2 ASPR and age-standardized YLD rate with percentage changes of anemia in China, 1990−2023. Fig. S1 Annual trends in ASPR and age-standardized YLD rate for anemia. Fig. S2 Annual trends in ASPR and age-standardized YLD rate for mild anemia (a), moderate anemia (b), and severe anemia (c). Fig. S3 Annual trends in age-standardized prevalence rate (ASPR) for anemia in China. Fig. S4 Annual trends in age-standardized YLD rate for anemia in China. Fig. S5 Annual trends in age-standardized prevalence rate (ASPR) for mild anemia in China. Fig. S6 Annual trends in age-standardized YLD rate for mild anemia in China. Fig. S7 Annual trends in age-standardized prevalence rate (ASPR) for moderate anemia in China. Fig. S8 Annual trends in age-standardized YLD rate for moderate anemia in China. Fig. S9 Annual trends in age-standardized prevalence rate (ASPR) for severe anemia in China. Fig. S10 Annual trends in age-standardized YLD rate for severe anemia in China. Fig. S11 The numbers with prevalence rates and YLD rates of anemia in China in 2023. Fig. S12 Number and rate due to anemia per 100,000 population attributable to each underlying cause by age in China in 2023. Fig. S13 Increment in anemia prevalence and YLD due to the changes in population growth, population aging, and age-specific prevalence rate in China from 1990 to 2023. Fig. S14 The association between SDI and the age-standardized prevalence rate (ASPR) and age-standardized YLD rate of anemia in China. [file 40779_2025_681_MOESM1_ESM.pdf]

Table S1 Hemoglobin concentration thresholds (g/L) for classification of anemia severity by stratification variables: sex, age, and pregnancy status

Stratification variables	Hemoglobin concentration thresholds (g/L)		
	**Mild anemia (100 − 159)**	**Moderate anemia (70 − 144)**	**Severe anemia (< 100)**
**Male**			
0 – 6 d	145 – 159	100 – 144	< 100
7 – 27 d	120 – 134	85 – 119	< 85
1 month – 4 years	100 – 109	70 – 99	< 70
5 – 14 years	110 – 114	80 – 109	< 80
≥ 15 years	110 – 129	80 – 109	< 80
**Female**			
Age			
0 – 6 d	145 – 159	100 – 144	< 100
7 – 27 d	120 – 134	85 – 119	< 85
1 month – 4 years	100 – 109	70 – 99	< 70
5 – 14 years	110 – 114	80 – 109	< 80
≥ 15 years			
Pregnancy status			
Non-pregnant	110 – 119	80 – 109	< 80
Pregnant	100 – 109	70 – 99	< 70

Table S2 ASPR and age-standardized YLD rate with percentage changes of anemia in China, 1990 − 2023

Parameter	ASPR			Age-standardized YLD rate		
	1990 (per 100,000, 95% UI)	2023 (per 100,000, 95% UI)	Change (%, 95% UI)	1990 (per 100,000, 95% UI)	2023 (per 100,000, 95% UI)	Change (%, 95% UI)
**China**	26,866.94 (21,633.81 − 33,347.99)	11,073.56 (9252.29 − 14535.13)	-58.78 (-70.21 to -45.79)	671.08 (427.87 − 1065.39)	242.36 (147.16 − 376.59)	-63.88 (-75.89 to -47.02)
Anhui	26,702.36 (21,499.20 − 33,114.58)	9850.61 (8360.76 − 11,614.24)	-63.11 (-73.32 to -51.05)	690.32 (445.00 − 1080.45)	218.45 (132.06 − 343.07)	-68.36 (-79.55 to -53.72)
Beijing	24,348.63 (20,311.05 − 29,662.08)	10,454.94 (8012.68 − 16,142.50)	-57.06 (-68.90 to -36.42)	592.87 (389.10 − 893.85)	224.14 (119.50 − 412.87)	-62.19 (-77.53 to -39.31)
Chongqing	28,981.72 (23,529.26 − 36,163.73)	11,365.81 (9091.76 − 17,302.22)	-60.78 (-71.48 to -41.56)	756.53 (477.89 − 1189.36)	244.82 (147.77 − 389.84)	-67.64 (-78.80 to -52.12)
Fujian	25,553.99 (20,532.45 − 31,516.65)	9251.68 (7718.84 − 11,407.34)	-63.80 (-73.74 to -52.87)	629.44 (395.23 − 995.84)	199.59 (121.24 − 317.53)	-68.29 (-78.88 to -52.61)
Gansu	30,159.69 (24,172.56 − 38,436.34)	13,831.73 (10,818.70 − 24,787.55)	-54.14 (-67.27 to -21.20)	754.86 (474.45 − 1193.76)	291.70 (172.24 − 483.23)	-61.36 (-75.26 to -42.07)
Guangdong	25,668.87 (20,605.13 − 31895.34)	9240.45 (7825.28 − 11,200.86)	-64.00 (-73.96 to -52.86)	652.88 (422.77 − 1016.55)	203.17 (120.63 − 318.27)	-68.88 (-79.50 to -52.21)
Guangxi	25442.46 (20335.51 − 31,525.25)	10,474.13 (8766.73 − 12,532.56)	-58.83 (-70.20 to -46.40)	616.49 (386.79 − 974.57)	227.09 (136.96 − 359.80)	-63.16 (-75.87 to -45.62)
Guizhou	30,779.58 (25,299.87 − 38,158.13)	13,026.39 (10,678.41 − 17,406.67)	-57.68 (-69.17 to -44.67)	769.28 (480.83 − 1236.63)	280.37 (169.86 − 443.73)	-63.55 (-75.84 to -46.52)
Hainan	25,005.46 (19,279.55 − 33,821.97)	15,043.06 (7398.16 − 42,069.06)	-39.84 (-71.00 to 48.99)	580.46 (365.79 − 897.73)	311.54 (123.53 − 777.35)	-46.33 (-77.16 to 17.39)
Hebei	23,887.48 (19,067.41 − 29,612.53)	11,563.17 (9790.12 − 13,675.32)	-51.59 (-65.29 to -34.92)	574.65 (366.84 − 905.40)	254.71 (152.80 − 397.37)	-55.68 (-70.98 to -34.13)
Heilongjiang	25,704.25 (20,574.89 − 31,744.17)	11,776.41 (9762.25 − 15,402.19)	-54.18 (-66.91 to -39.37)	624.18 (391.62 − 981.60)	257.12 (156.15 − 406.67)	-58.81 (-72.74 to -39.40)

Parameter	ASPR			Age-standardized YLD rate		
	1990 (per 100,000, 95% UI)	2023 (per 100,000, 95% UI)	Change (%, 95% UI)	1990 (per 100,000, 95% UI)	2023 (per 100,000, 95% UI)	Change (%, 95% UI)
Henan	25,605.56 (20,439.96 − 32,010.55)	10694.93 (9037.56 − 12,771.72)	-58.23 (-70.22 to -44.29)	632.61 (405.39 − 993.28)	234.73 (141.54 − 370.66)	-62.89 (-75.77 to -44.14)
Hong Kong	21,302.20 (17,396.82 − 25,352.99)	13,897.31 (10,512.58 − 18,679.73)	-34.76 (-50.91 to -13.06)	527.69 (307.97 − 822.58)	360.77 (186.84 − 695.72)	-31.63 (-54.94 to 21.42)
Hubei	27,060.12 (21,636.57 − 33,854.84)	9912.06 (8353.39 − 11,716.32)	-63.37 (-73.72 to -51.08)	680.25 (433.80 − 1071.01)	215.01 (130.51 − 341.58)	-68.39 (-79.34 to -53.76)
Hunan	27,075.70 (21,613.46 − 33,955.74)	10,172.65 (8555.69 − 11,928.81)	-62.43 (-73.23 to -50.10)	668.91 (426.24 − 1057.71)	218.89 (132.18 − 353.75)	-67.28 (-78.95 to -52.84)
Inner Mongolia	30,606.40 (24,668.77 − 38,720.14)	14,202.01 (11,067.27 − 25,651.03)	-53.60 (-66.92 to -22.58)	788.30 (496.71 − 1236.48)	305.35 (181.88 − 499.08)	-61.26 (-74.92 to -42.47)
Jiangsu	25,625.26 (20,609.69 − 31,707.46)	9910.52 (8406.62 − 11,814.48)	-61.33 (-71.83 to -49.28)	658.89 (425.22 − 1027.44)	221.20 (134.57 − 346.58)	-66.43 (-77.69 to -49.87)
Jiangxi	27,052.22 (21,792.32 − 33,541.06)	9462.13 (7957.31 − 11,142.04)	-65.02 (-74.63 to -54.69)	687.25 (435.73 − 1079.75)	206.61 (123.57 − 324.62)	-69.94 (-80.14 to -56.04)
Jilin	27,466.30 (22,106.42 − 34,368.91)	11,644.79 (9182.37 − 18,856.97)	-57.60 (-69.75 to -29.65)	670.02 (418.04 − 1070.37)	243.59 (147.43 − 395.15)	-63.64 (-76.38 to -46.15)
Liaoning	24,154.62 (19,336.24 − 29,654.56)	11,417.61 (9563.68 − 13,551.43)	-52.73 (-65.54 to -37.95)	590.70 (367.03 − 928.93)	254.59 (153.34 − 397.50)	-56.90 (-71.58 to -36.16)
Macao	16,506.03 (13,095.88 − 20,242.72)	8260.50 (6930.09 − 9906.03)	-49.95 (-63.44 to -34.60)	364.16 (229.07 − 578.89)	180.79 (108.51 − 293.12)	-50.35 (-67.08 to -29.46)
Ningxia	32,058.09 (25,176.05 − 44,338.98)	16,193.75 (8256.34 − 49,926.34)	-49.49 (-74.08 to 45.70)	797.25 (509.72 − 1240.64)	349.94 (158.80 − 879.43)	-56.11 (-78.48 to -11.22)
Qinghai	24,682.85 (19,710.44 − 31,428.69)	14,442.08 (7086.45 − 35,414.27)	-41.49 (-70.90 to 46.94)	657.16 (407.67 − 1037.16)	365.83 (132.74 − 956.42)	-44.33 (-78.70 to 30.93)
Shaanxi	29,710.10 (24,356.42 − 36,719.53)	11,579.35 (9566.32 − 15291.00)	-61.03 (-71.71 to -49.69)	732.23 (454.83 − 1166.81)	244.87 (148.31 − 389.51)	-66.56 (-77.90 to -50.79)

Parameter	ASPR			Age-standardized YLD rate		
	1990 (per 100,000, 95% UI)	2023 (per 100,000, 95% UI)	Change (%, 95% UI)	1990 (per 100,000, 95% UI)	2023 (per 100,000, 95% UI)	Change (%, 95% UI)
Shandong	25,578.27 (20,522.31 − 31,792.11)	10,757.96 (9143.90 − 12,986.55)	-57.94 (-69.89 to -43.79)	644.61 (413.35 − 1010.52)	240.26 (144.32 − 376.75)	-62.73 (-75.73 to -43.88)
Shanghai	27,765.43 (23,963.65 − 32,261.77)	12,922.97 (9840.48 − 22,458.47)	-53.46 (-66.60 to -22.54)	713.13 (464.05 − 1079.53)	294.26 (163.71 − 522.22)	-58.74 (-73.65 to -39.08)
Shanxi	31,470.70 (26,028.16 − 38,227.21)	14,404.68 (11,910.94 − 19,169.62)	-54.23 (-65.68 to -41.96)	821.00 (516.58 − 1313.32)	321.17 (194.70 − 503.73)	-60.88 (-73.77 to -42.62)
Sichuan	28,657.82 (22,738.46 − 36,474.09)	11,587.15 (9779.04 − 13,897.27)	-59.57 (-71.73 to -45.70)	697.92 (444.88 − 1098.26)	246.22 (147.92 − 386.24)	-64.72 (-76.62 to -45.42)
Tianjin	24,590.78 (19,789.61 − 30,977.30)	12,672.43 (8142.49 − 26,858.90)	-48.47 (-67.51 to 8.36)	620.98 (387.87 − 980.42)	291.72 (138.86 − 586.33)	-53.02 (-74.59 to -16.71)
Xizang	14,344.34 (11,410.24 − 17,821.84)	8796.09 (4804.64 − 17,317.33)	-38.68 (-66.57 to 22.95)	360.07 (221.04 − 572.51)	246.89 (82.56 − 618.65)	-31.43 (-73.57 to 79.82)
Xinjiang	30,626.51 (24,541.17 − 38,642.96)	15,722.56 (11,723.60 − 32,467.30)	-48.66 (-63.59 to -3.98)	773.23 (479.29 − 1225.00)	335.71 (201.30 − 557.34)	-56.58 (-71.94 to -35.73)
Yunnan	30,146.13 (24,100.32 − 38,104.70)	13,335.07 (11,165.93 − 16,183.95)	-55.77 (-68.34 to -40.84)	752.79 (476.89 − 1186.26)	289.85 (177.34 − 461.15)	-61.50 (-74.99 to -41.76)
Zhejiang	26,361.33 (21,344.22 − 32,592.09)	9853.33 (8317.60 − 11,663.85)	-62.62 (-72.63 to -51.88)	674.90 (428.14 − 1056.22)	216.92 (130.94 − 341.78)	-67.86 (-78.73 to -53.08)

ASPR age-standardized prevalence rate, *YLD* years lived with disability, *UI* uncertainty interval

Figure legends

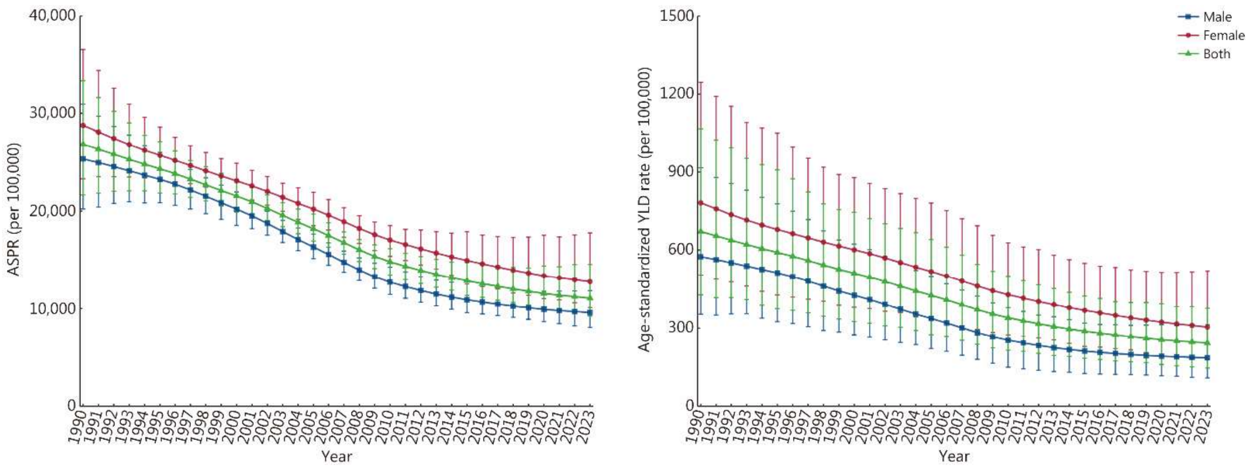

Fig. S1 Annual trends in ASPR and age-standardized YLD rate for anemia. ASPR age-standardized prevalence rate, YLD years lived with disability

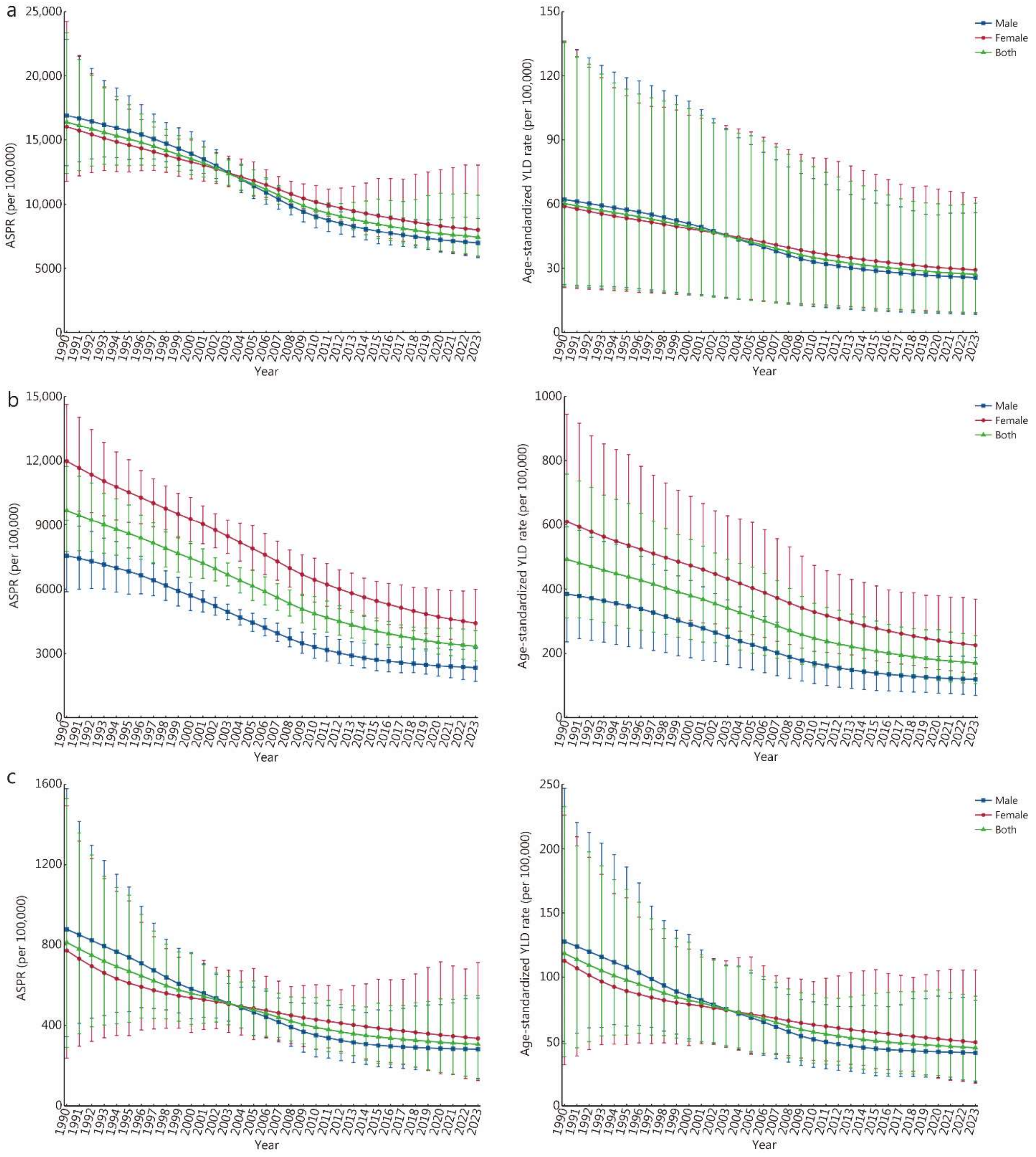

Fig. S2 Annual trends in ASPR and age-standardized YLD rate for mild anemia (**a**), moderate anemia (**b**), and severe anemia (**c**). ASPR age-standardized prevalence rate, YLD years lived with disability

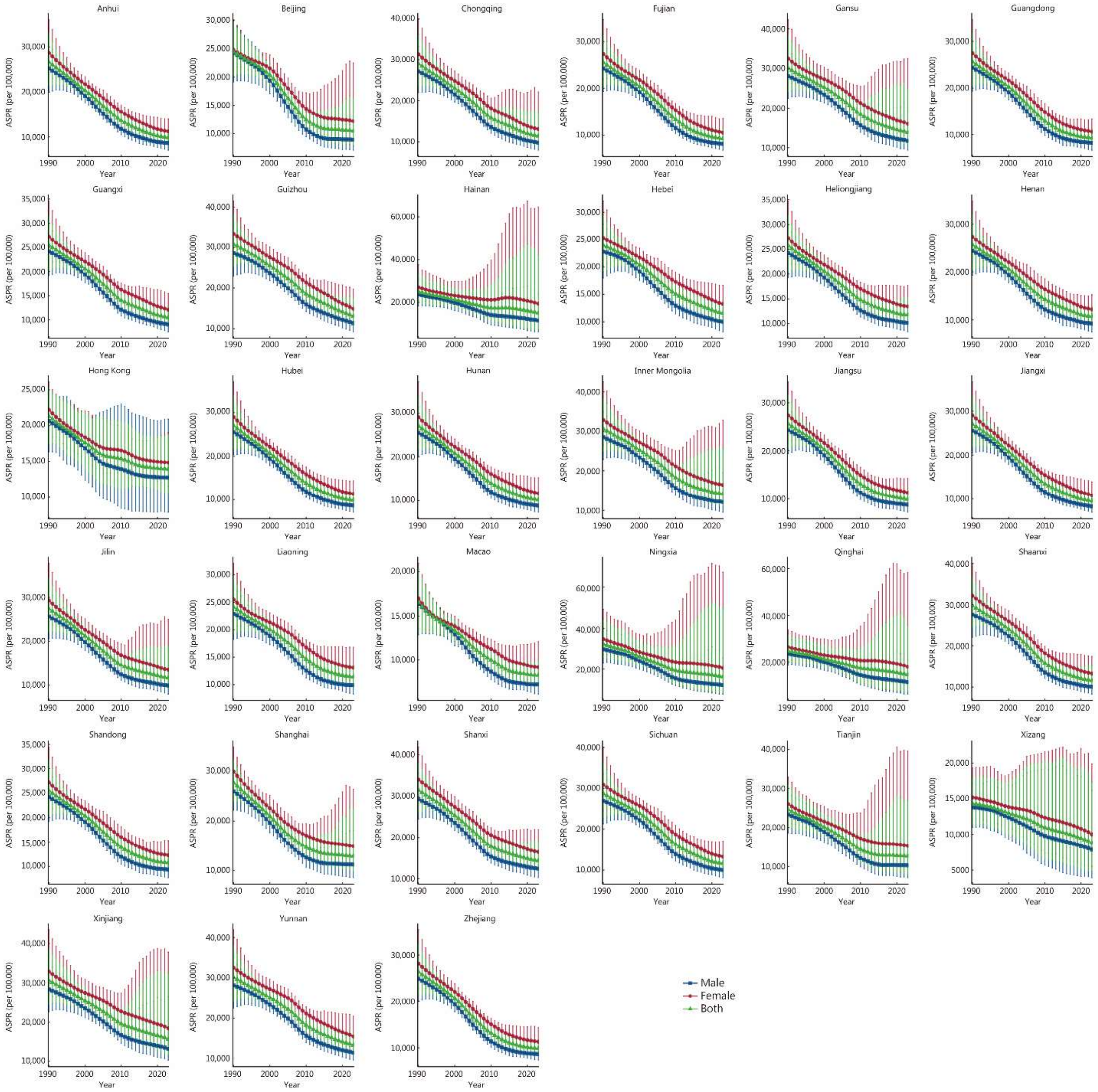

Fig. S3 Annual trends in age-standardized prevalence rate (ASPR) for anemia in China

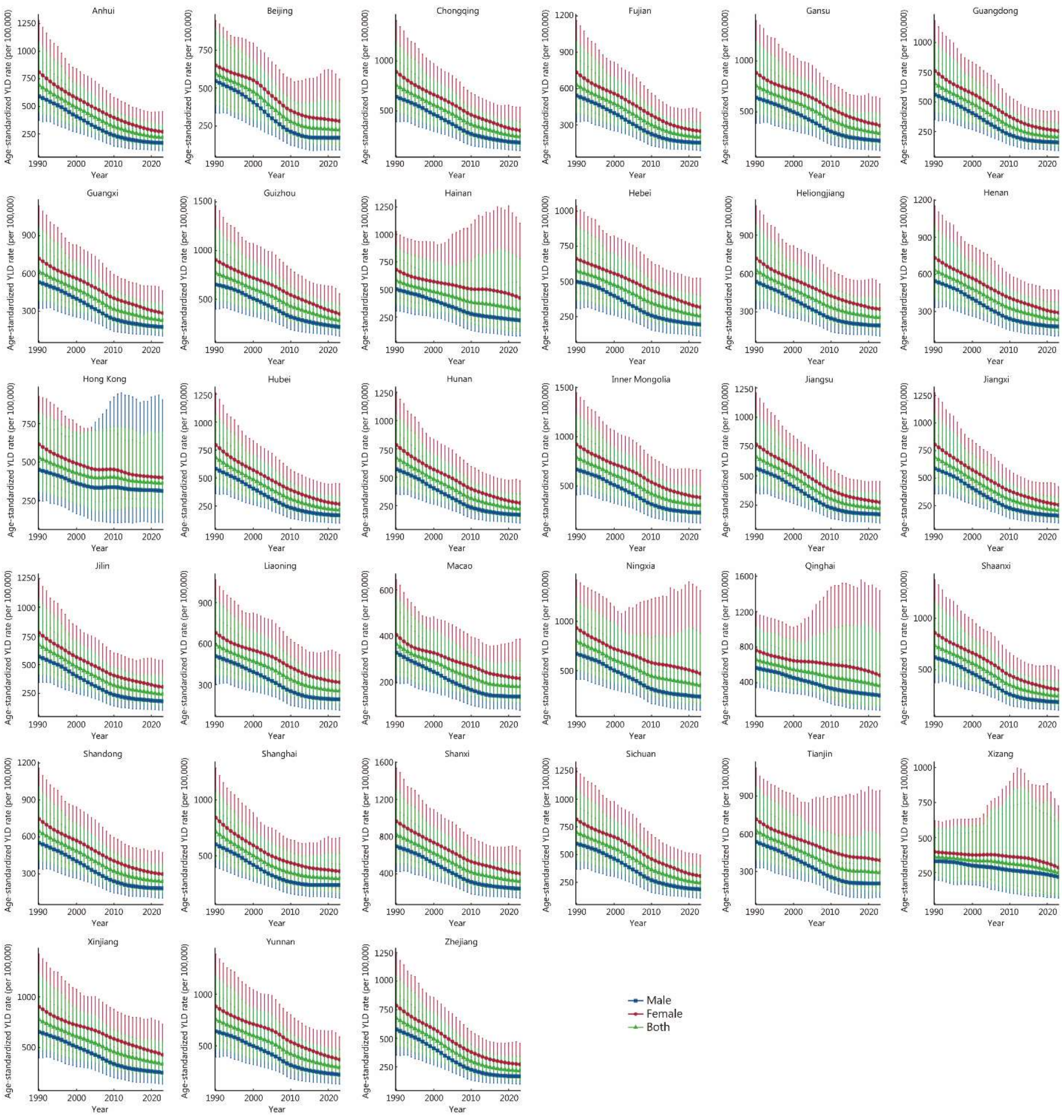

Fig. S4 Annual trends in age-standardized YLD rate for anemia in China. YLD years lived with disability

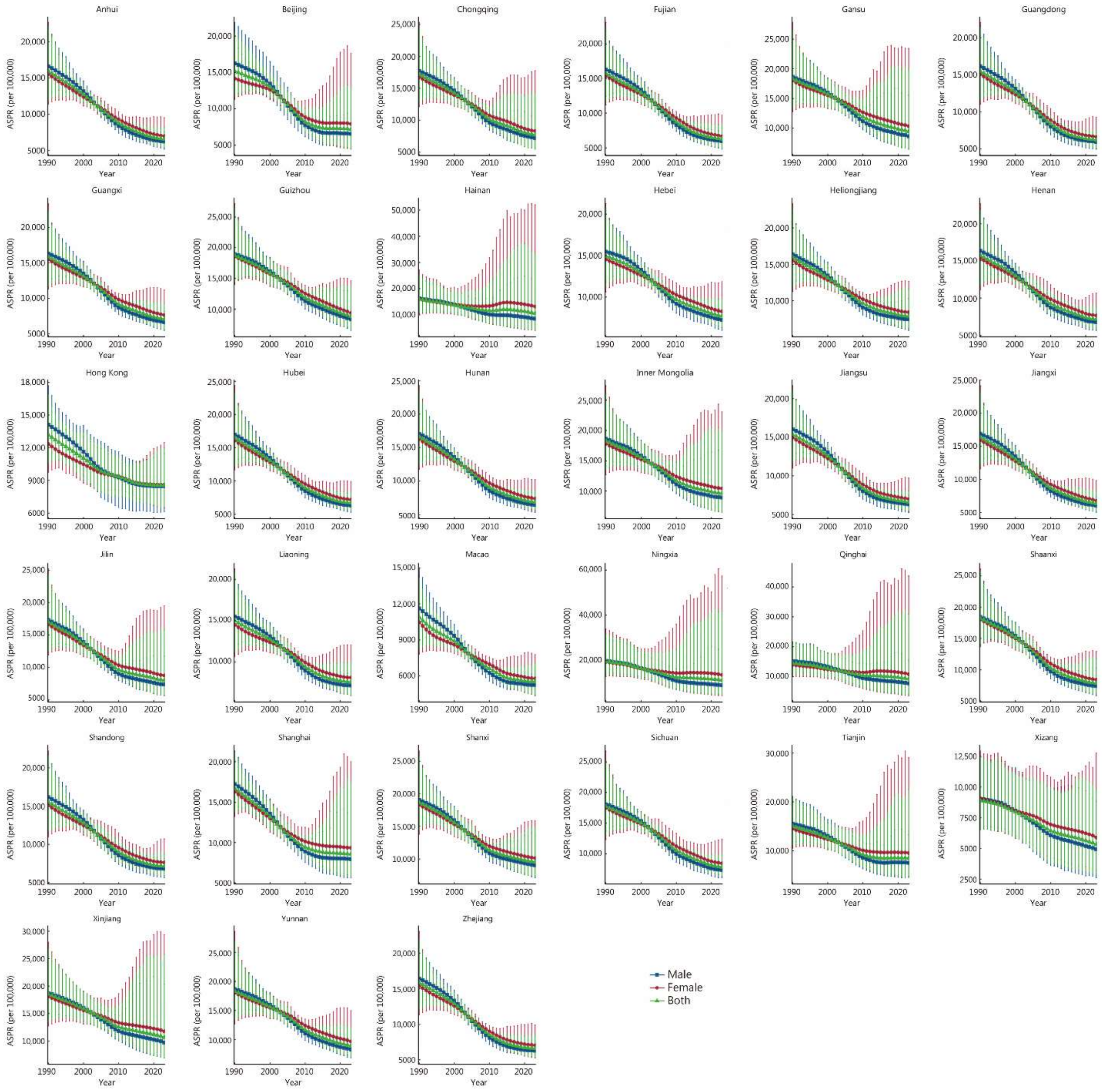

Fig. S5 Annual trends in age-standardized prevalence rate (ASPR) for mild anemia in China

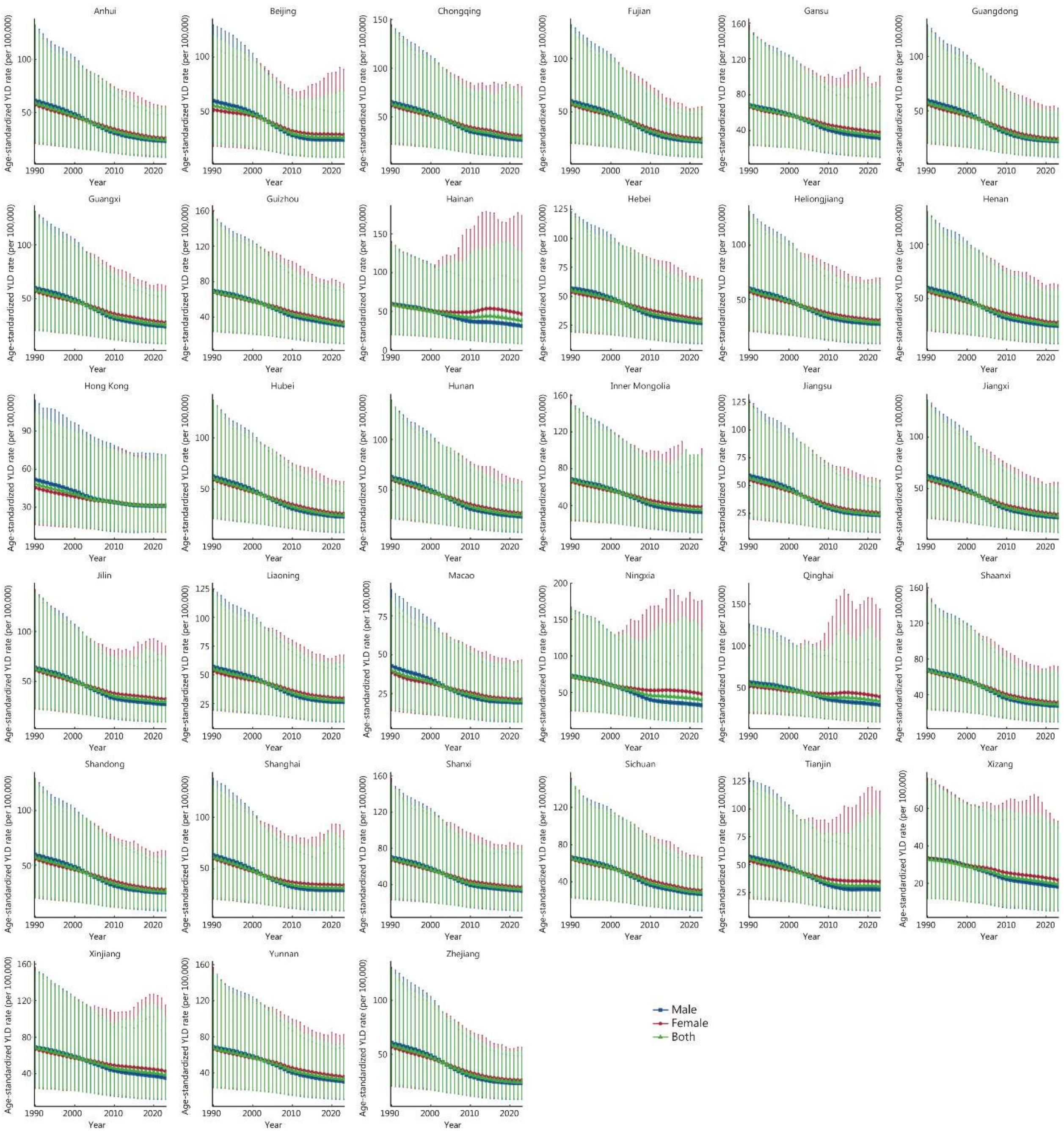

Fig. S6 Annual trends in age-standardized YLD rate for mild anemia in China. YLD years lived with disability

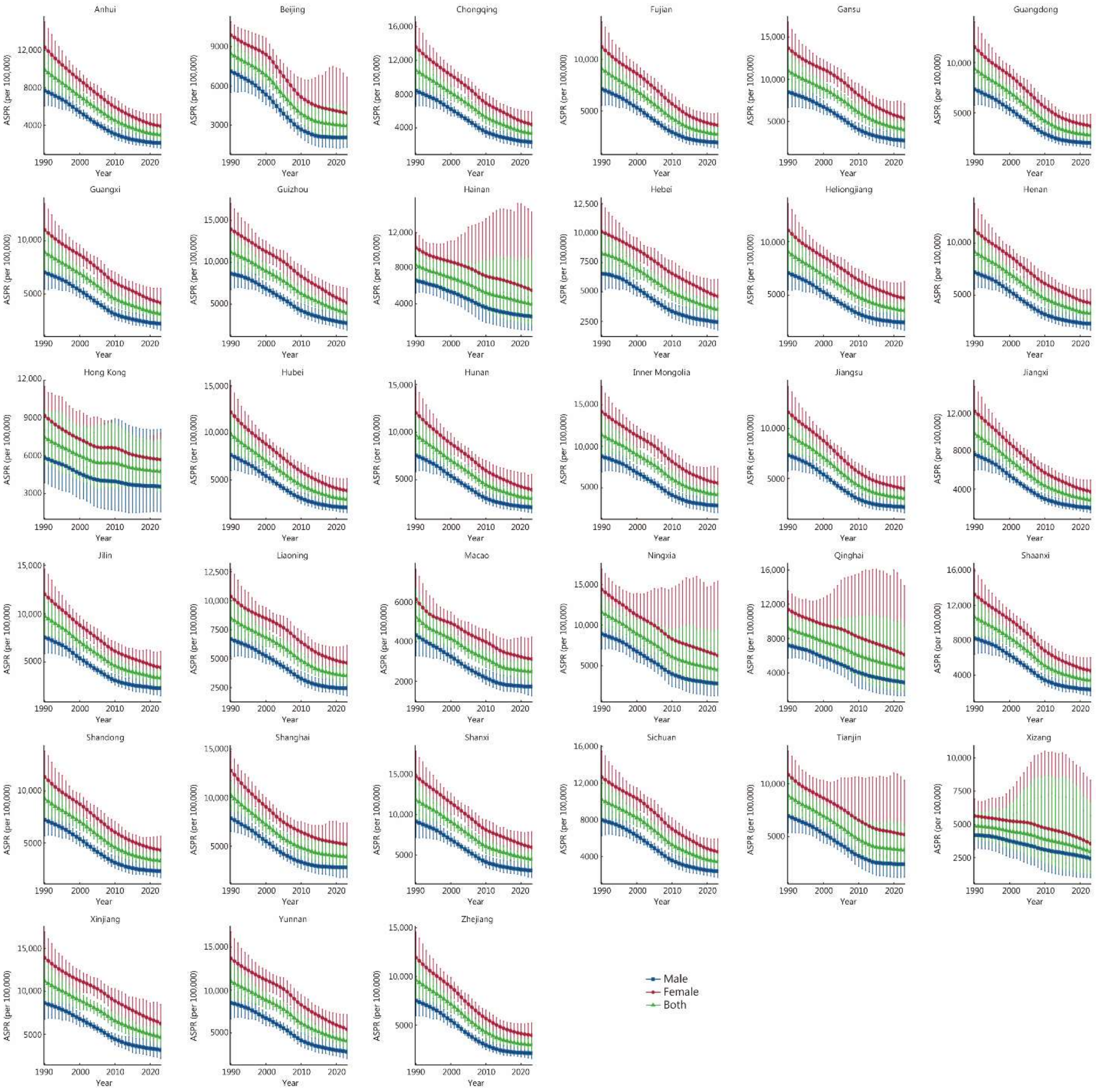

Fig. S7 Annual trends in age-standardized prevalence rate (ASPR) for moderate anemia in China

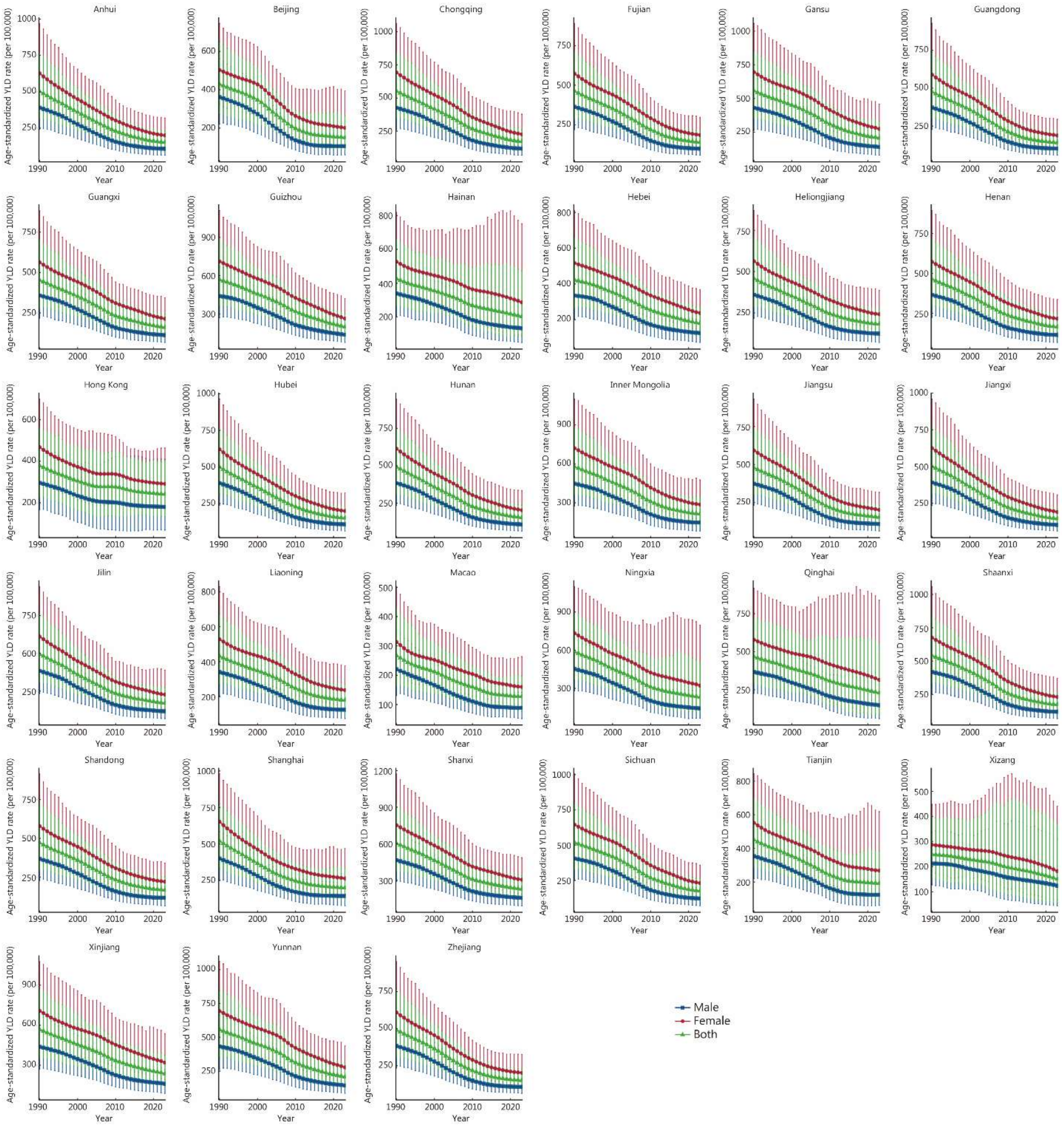

Fig. S8 Annual trends in age-standardized YLD rate for moderate anemia in China. YLD years lived with disability

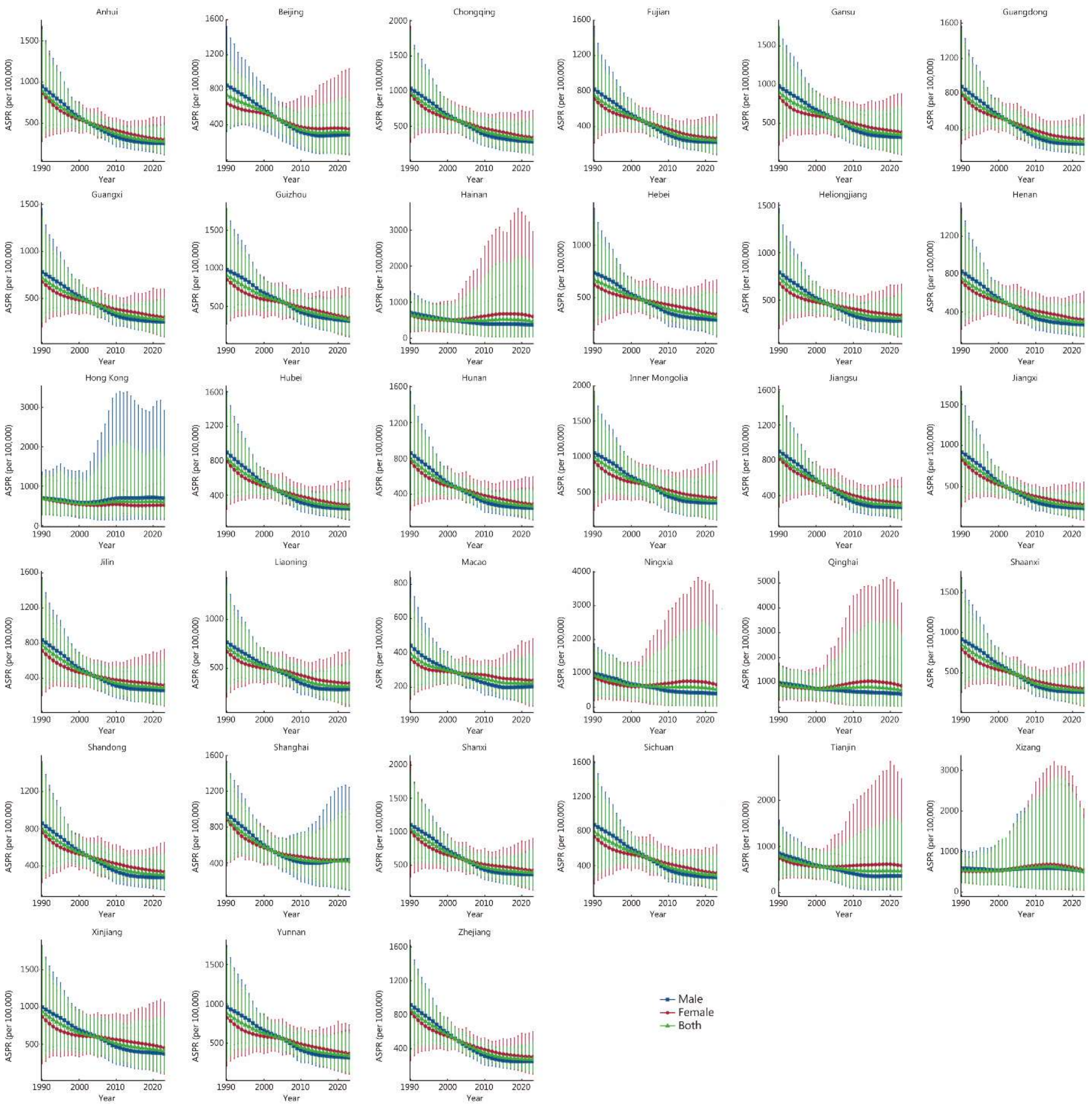

Fig. S9 Annual trends in age-standardized prevalence rate (ASPR) for severe anemia in China

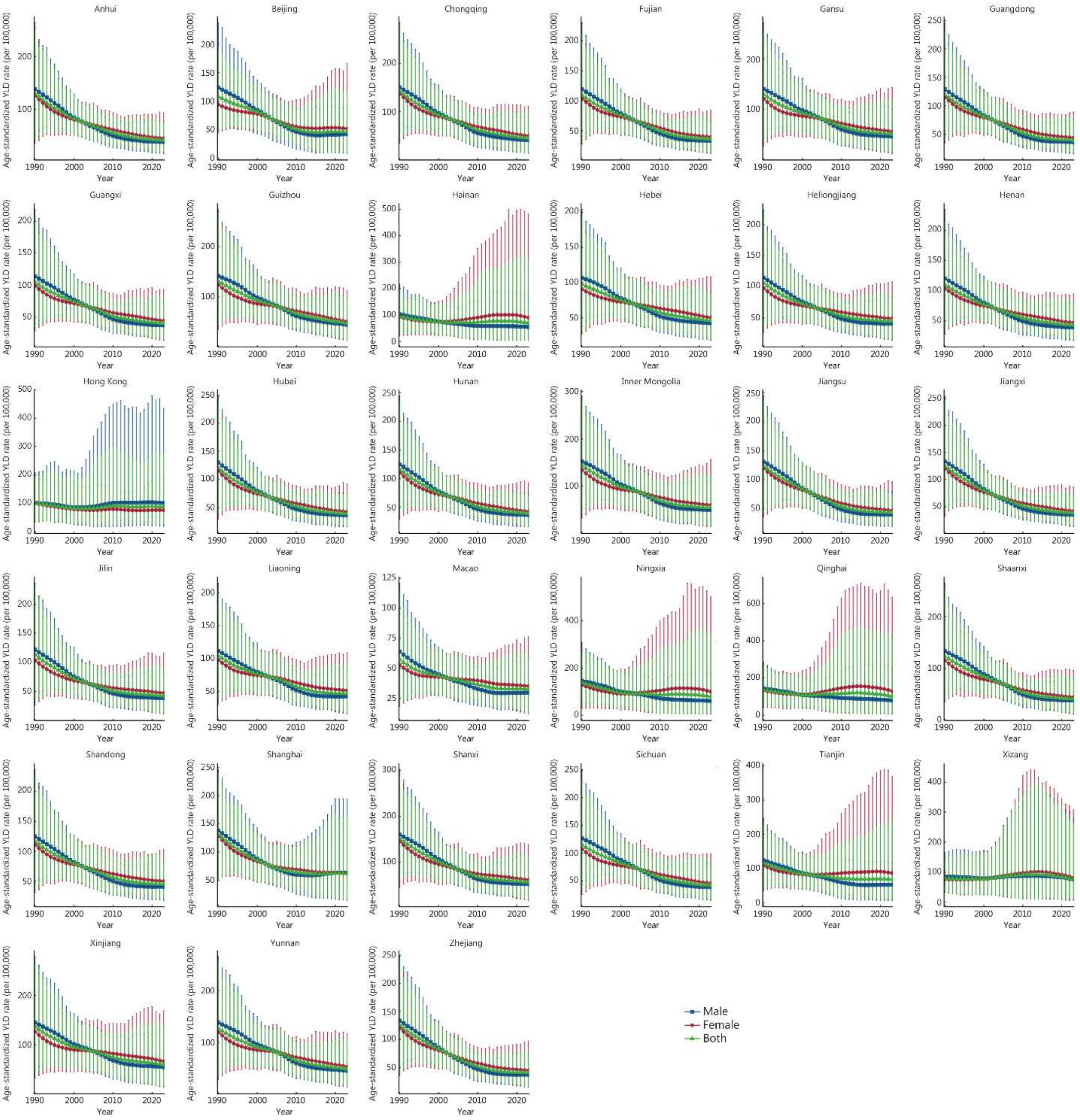

Fig. S10 Annual trends in age-standardized YLD rate for severe anemia in China. YLD years lived with disability

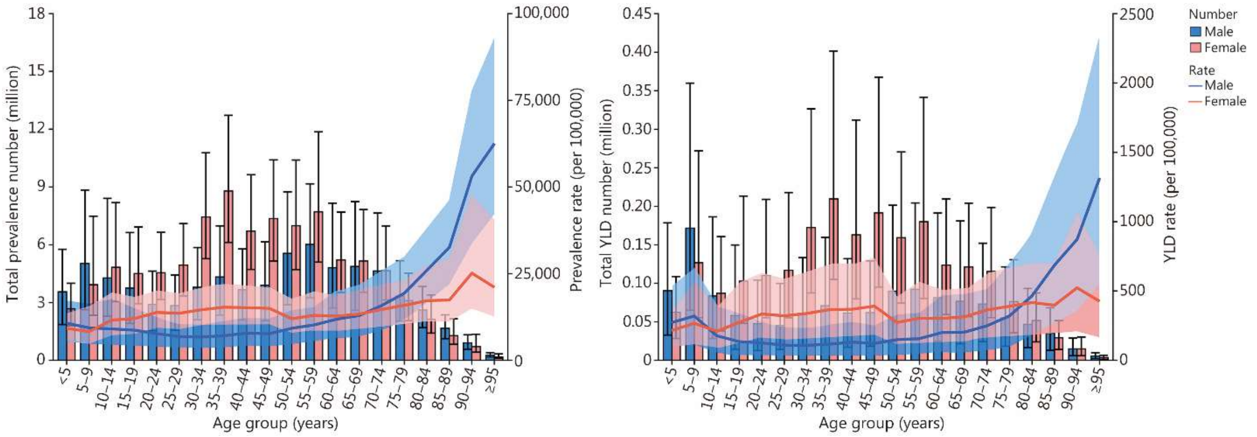

Fig. S11 The numbers with prevalence rates and YLD rates of anemia in China in 2023. YLD years lived with disability

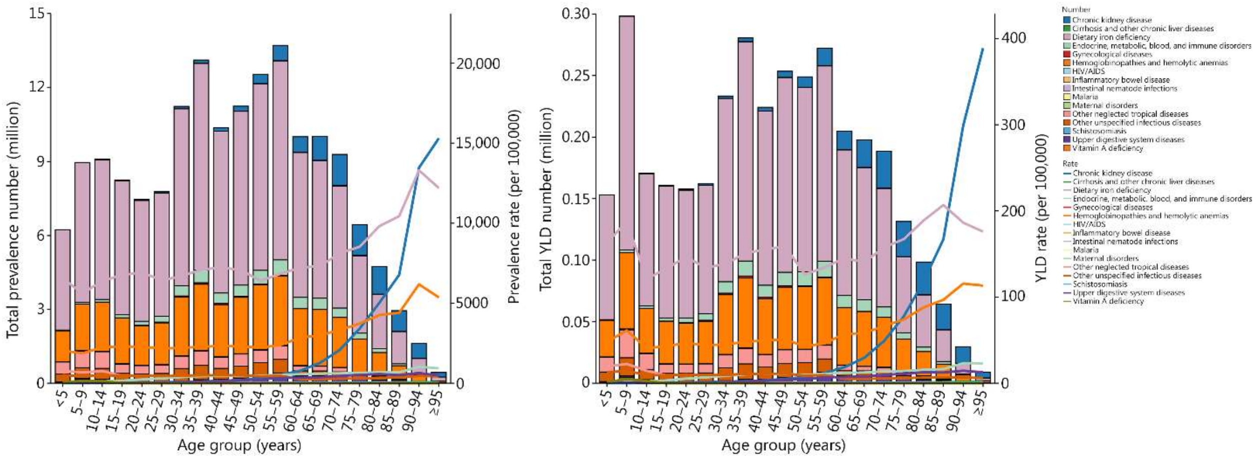

Fig. S12 Number and rate due to anemia per 100,000 population attributable to each underlying cause by age in China in 2023. YLD years lived with disability, HIV/AIDS human immunodeficiency virus/acquired immunodeficiency syndrome

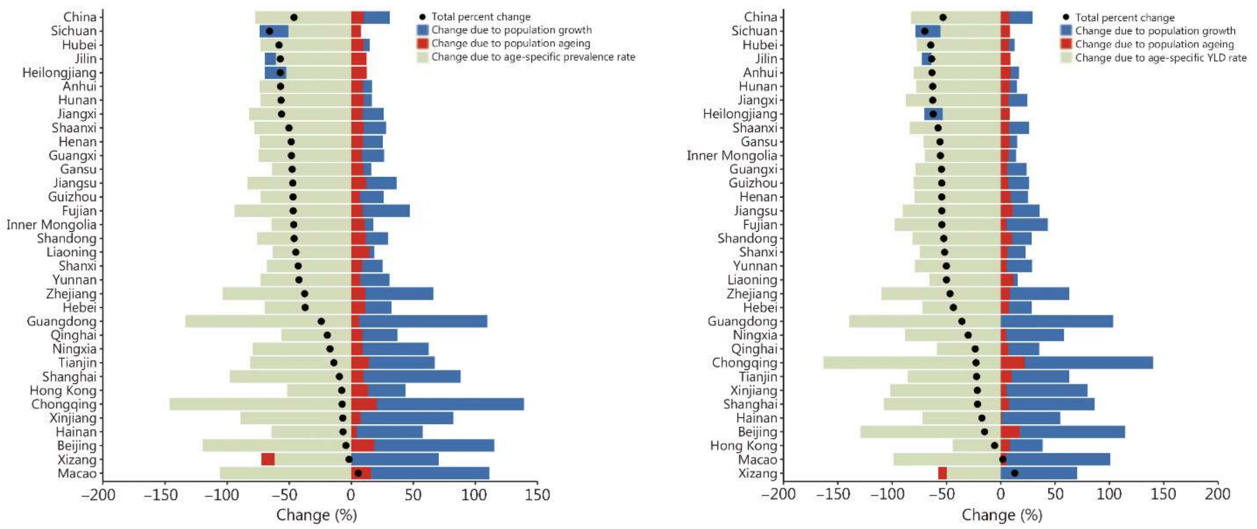

Fig. S13 Increment in anemia prevalence and YLD due to the changes in population growth, population aging, and age-specific prevalence rate in China from 1990 to 2023. YLD years lived with disability

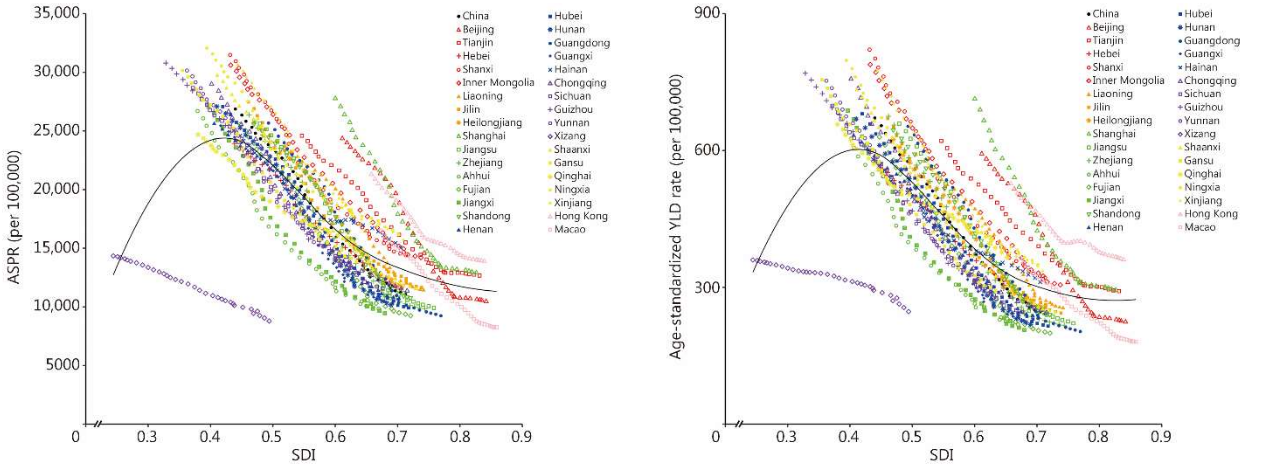

Fig. S14 The association between SDI and the age-standardized prevalence rate (ASPR) and age-standardized YLD rate of anemia in China. Black line locally weighted regression curve. SDI socio-demographic index, ASPR age-standardized prevalence rate, YLD years lived with disability
